# Antibody-Recruiting Surfaces Using Adaptive Multicomponent Supramolecular Copolymers

**DOI:** 10.1021/acs.biomac.5c00043

**Published:** 2025-04-09

**Authors:** Marle E. J. Vleugels, Esmee de Korver, Simone I. S. Hendrikse, Sinan Kardas, Shikha Dhiman, Bas F. M de Waal, Sandra M. C. Schoenmakers, Stefan Wijker, Bruno G. De Geest, Mathieu Surin, Anja R. A. Palmans, E. W. Meijer

**Affiliations:** † Laboratory of Macromolecular and Organic Chemistry, Institute for Complex Molecular Systems, Eindhoven University of Technology, P.O. Box 513, Eindhoven 5600 MB, The Netherlands; ‡ Laboratory for Chemistry of Novel Materials, Center of Innovation and Research in Materials and Polymers, University of Mons−UMONS, Mons 7000, Belgium; § Department of Pharmaceutics, Ghent University, Ghent 9000, Belgium; ∥ School of Chemistry and RNA Institute, UNSW, Sydney, New South Wales 2052, Australia

## Abstract

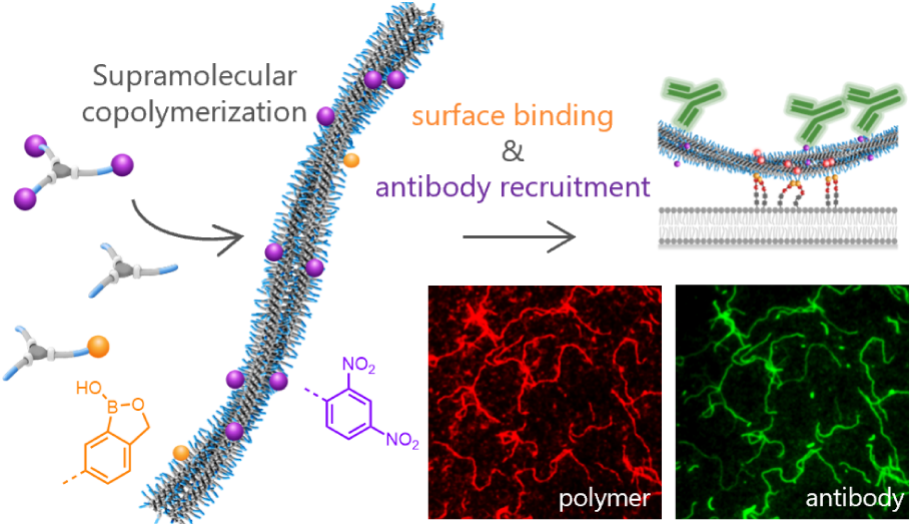

Multicomponent structures that mediate the clustering of antibodies on cancer cell surfaces are an attractive strategy to unleash innate immune killing mechanisms. However, covalent multifunctional scaffolds that combine cell surface anchoring and antibody binding can be challenging to synthesize and lack adaptability. Here, we present a dynamic multicomponent supramolecular system displaying both antibody- and cell surface-binding motifs, without covalent linkage between them. Supramolecular monomers based on benzene-1,3,5-tricarboxamide (**BTA-(OH)**_3_) were functionalized with benzoxaborole (Ba) for surface anchoring (**BTA-Ba**) or dinitrophenyl (DNP) for antibody binding (**BTA-DNP**_**1/3**_). The multicomponent fibers comprising **BTA-(OH)**_**3**_, **BTA**-**Ba**, and **BTA-DNP**_**1/3**_ recruited anti-DNP antibodies to sialic acid-functionalized supported lipid bilayers, indicating that both Ba and DNP remained accessible for binding. Dynamic exchange was demonstrated in a cell-mimicking environment, highlighting the adaptivity of these supramolecular polymers. Despite the complexity of a ternary system, the adaptivity of supramolecular polymers gives the individual components the possibility to act in concert, mimicking natural systems.

## Introduction

Monoclonal antibody (mAb) therapeutics have emerged over the past years as an important strategy for cancer treatment.^1^ The mechanism of action of several mAbs relies on the binding and clustering of antibodies on pathogen cell surfaces, which marks the cells for destruction by the immune system. Despite their widespread use, the production of monoclonal antibodies comes with high costs and undesired immunogenicity can arise.^2^ Alternatively, the utilization of endogenous antibodies already present in the bloodstream would eliminate the need to externally administer mAbs.^3^ Small molecule ligands known to interact with these antibodies include galactose-α-1,3-galactose, phosphorylcholine, rhamnose, and dinitrophenyl (DNP).^3^ Antibody-recruiting molecules (ARMs), which are bifunctional small molecules comprising a cell-anchoring and an antibody-binding motif, liaise the recruitment of endogenous antibodies to cell surfaces.^3,4^ To increase antibody recruitment toward cell surfaces, the creation of structures that present multiple copies of the antibody-binding motif is of interest, as this could lead to enhanced binding due to the multivalent effect.^5^ Enhanced binding due to multivalency has already been demonstrated in the literature on polymeric^6−8^ and dendritic^9−11^ constructs. However, the spacing and geometry of the antibody-binding motif has an influence on the binding capabilities,^12,13^ whereas the covalent nature of these constructs restricts their adaptiveness.

Nature utilizes supramolecular structures to construct both multivalent and multifunctional scaffolds, such as the 1D fibrillar structures found in the extracellular matrix.^14^ Due to the noncovalent interactions between the monomers, e.g., hydrophobic interactions, hydrogen bonding, electrostatic interactions, or π–π stacking, the resulting structures are intrinsically dynamic and adaptive. This dynamic adaptivity is key for rearrangements to occur and subsequent signal processing. In order to mimic these dynamic and adaptable structures found in nature, synthetic supramolecular polymers are promising candidates.^15,16^ Recent studies have highlighted the importance of dynamicity in the interaction of synthetic fibrillar structures with cellular receptors.^17,18^ By introducing a specific functionality in the peptide block of peptide amphiphiles, such as sulfonated saccharides, binding to growth factors and an increase in signaling were demonstrated.^19,20^ A similar strategy to bind growth factor proteins was also explored using ureidopyrimidinone (UPy)-based assemblies.^21^ Moreover, protein binding and protein assembly along supramolecular fibers have been achieved with *C*_3_-symmetric discotic molecules by introducing biotinylated ligands.^22^

**Figure 1:**
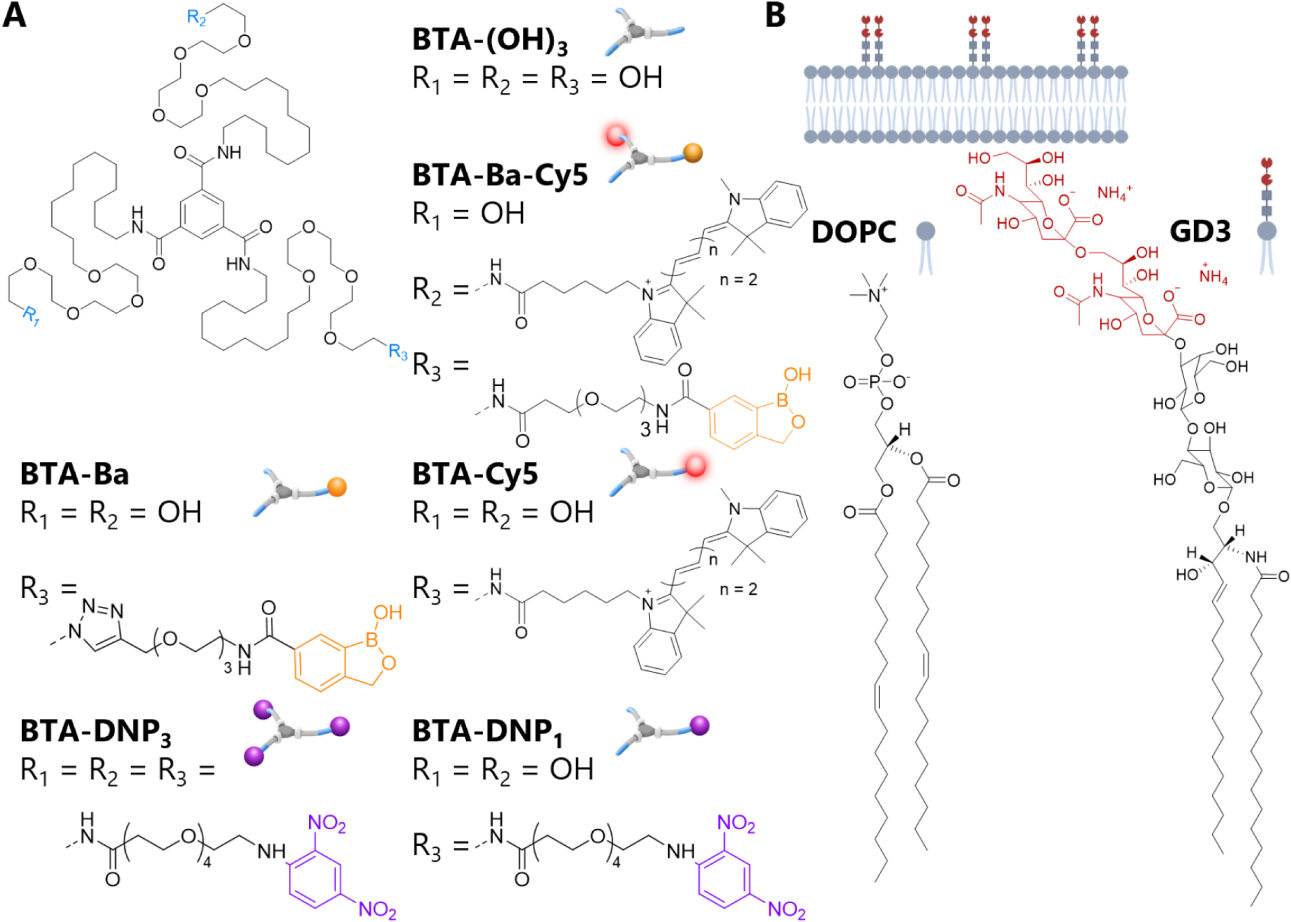
(A) Chemical structures of the BTA-based monomers used in this study. (B) Chemical structures of the lipids composing the supported lipid bilayer (SLB, cartoon on top).

A well-studied supramolecular motif capable of forming fibrillar structures in water is benzene-1,3,5-tricarboxamide (**BTA-(OH)**_3_) (Figure 1), where structure formation is driven by hydrophobic interactions, π–π stacking, and hydrogen bonding between the amides.^23,24^**BTA-(OH)**_**3**_ forms dynamic one-dimensional fibers consisting of two monomers in the cross-section, which form a double-helical structure.^25−27^ With this platform, the number and nature of functional groups can be easily tuned via supramolecular copolymerization. The introduction of functional groups at the periphery of these supramolecular polymers has been studied at a fundamental level and includes the use of short peptides^28^ and small saccharides.^29−31^ In addition, protein recruitment has been achieved by introducing DNA linkers.^32^ Recently, the first studies toward biomedical applications were conducted that explored BTA-based systems for cell–material interactions. The introduction of benzoxaborole (Ba) provided a specific interaction with sialic acid units present on the surface of human red blood cells.^33^ Pathogen inhibition and increased binding affinity were achieved by the introduction of a small, cationic protein inhibitor.^34^ In both studies, low concentrations of the functional BTA-based monomer were sufficient to observe an increase in binding affinity, underlining the role of multivalency in cell–material interactions. Additionally, a recent study on dynamic multicomponent systems focusing on the clustering of ligands anchored to the supramolecular polymers and receptors incorporated in supported lipid bilayers revealed reciprocity in dynamics between supramolecular fibers and a cell membrane mimic.^35^ To obtain a multicomponent, complex system that can exhibit multiple functions (i.e., cell binding and antibody recruitment), it is of great importance to understand the interplay between dynamicity and functionality at a fundamental level. However, the increase of complexity requires systems with three or more components, and these have been rarely studied.

Here, we integrate two functionalities into one BTA-based system: DNP for antibody binding and Ba for cell-surface binding, aiming for multivalent antibody recruitment. The dynamicity of the supramolecular copolymers was investigated with hydrogen/deuterium exchange followed by mass spectrometry (HDX-MS) while molecular dynamics (MD) simulations gave insight into the molecular packing and positioning of DNP units within the supramolecular polymers. The accessibility of the functional groups was investigated by surface anchoring of the supramolecular system on a supported lipid bilayer, followed by investigating the capability for antibody binding. Finally, the dynamicity of the supramolecular copolymers was investigated in an even more complex setting to mimic the cellular environment.

## Experimental Section

### Materials

Unless stated otherwise, all reagents and chemicals were obtained from commercial sources at the highest purity available and used without further purification. All solvents were of AR quality and purchased from BioSolve. Water was purified on an EMD Millipore Milli-Q Integral Water Purification System. Reactions were followed by thin-layer chromatography (precoated 0.25 mm, 60-F254 silica gel plates from Merck). Dry solvents were obtained with an MBRAUN Solvent Purification System (MB-SPS). Automated column chromatography was performed on a Biotage Isolera One column machine using Biotage Sfär or C18 cartridges. **BTA-(OH)**_**3**_, **BTA-(NH**_**2**_**)**_**3**_, **BTA-(NH**_**2**_**)**_**1**_, **BTA-NH**_**2**_**–N**_**3**_, **BTA-Ba**, **BTA-Cy5**, and **PFP-Ba** were synthesized according to the literature procedures.^23,26,28,33,34^

### Synthesis of Monomers

All details of the synthesis, purification, and characterization of the new monomers are given in the Supporting Information: **BTA-DNP**_**1**_ (LC-MS: calculated *m*/*z* = 1700 for C_86_H_153_N_7_O_26_, observed *m*/*z* = 568.08 [M+3H]^3+^, 851.50 [M+2H]^2+^, 1700.83 [M + H]^+^), **BTA-DNP**_**3**_ (MALDI-TOF-MS: calculated MW = 2525.99 g/mol for C_120_H_201_N_15_O_42_, observed *m*/*z* = 2548.47 [M + Na]^+^), and **BTA-Ba-Cy5** (LC-MS: Rt = 5.05 min, calculated *m*/*z* = 2125.40, calculated *m*/*z* for internal ester = 2108.40, observed *m*/*z* = 1069.42 [internal ester + MeOH + H]^2+^, 705.92 [internal ester +2H]^3+^, 529.75 [internal ester +3H]^4+^).

### Assembly of BTA Materials

#### Copolymerization

**BTA-(OH)**_**3**_ was copolymerized with functional monomers by weighing **BTA-(OH)**_**3**_ as a solid material into a glass vial and adding **BTA-DNP**_**1**_, BTA-DNP_3_, or **BTA-Ba** from a 500 μM stock solution in MeOH. The organic solvent was removed using an N_2_ (g) stream, after which MQ water was added to obtain the desired concentration. The sample was stirred at 80 °C for 15 min and vortexed for 15 s immediately afterward. All samples were left to equilibrate for 16–24 h at room temperature before being used for any measurements.

#### Copolymerization Including Dye-Labeled Molecules

Samples of the copolymers were prepared with the previously described protocol. After the heating and vortexing step, **BTA-Ba-Cy5** or **BTA-Cy5** was added from a 500 μM stock solution in MeOH at the desired concentration. The samples were then equilibrated at 45 °C for 15 min without stirring. The samples were left to equilibrate overnight at room temperature and then further equilibrated in the fridge for 1 week until the measurements to prevent degradation of the dye.

### Supported Lipid Bilayer Formation and Incubation of BTA Fibers

#### Substrate Cleaning and Preparation

8-well μ-slides were immersed in a 2 wt % sodium dodecyl sulfate (SDS) solution for 30 min, rinsed with demi water and sonicated in EtOH for 30 min, thoroughly rinsed with demineralized water, followed by Milli-Q water, and dried under a nitrogen stream. Activation was performed with a 15 min UV/ozone treatment.

#### Preparation of Small Unilamellar Vesicles (SUVs)

DOPC and GD3 were mixed in the desired molar ratios in a glass vial, from stock solutions in CHCl_3_ (DOPC, 10 mg/mL) or CHCl_3_/MeOH (GD3, 5 mg/mL). The solvent was evaporated with a nitrogen stream, and the lipidic film was dried for at least 1 h under high vacuum. The dried lipid film was resuspended in Milli-Q water to a concentration of 1 mg/mL. The lipid suspensions were extruded 11 times through a polycarbonate membrane (Whatman) with a 100 nm pore size. The obtained SUVs were stored in the refrigerator and used within a maximum of 2 weeks. For TIRF imaging of the vesicles, SUVs were labeled with Nile Red (5 mol %, from a 1 mM stock solution in ACN), diluted to 0.1 mg/mL in PBS (pH 7.4), and imaged in a homemade imaging chamber (see instrumentation).

#### Binding of BTA Fibers to the SLB

50 μM samples of BTA fibers in Milli-Q water were diluted to the appropriate concentration (2.5 or 1 μM) in PBS. Then, 200 μL of the BTA solution was added to the SLB. BTAs were incubated on the SLB for 1 h, after which unbound BTAs were removed from the well by washing three times with PBS.

#### Preparation of Supported Lipid Bilayers for TIRF

A vesicle fusion method was used to form the supported lipid bilayers (SLBs). SUVs were first diluted down to 0.1 mg/mL in 1X PBS. Before the formation of the SLB, 400 μL of an aqueous 2 M sodium hydroxide solution was added to the glass substrate for 1 h to form a hydrophilic surface. Afterward, the wells were rinsed with Milli-Q water three times and incubated with 200 μL of 0.1 mg/mL SUV solution for 30 min at room temperature. Excess lipids were removed from the wells by rinsing with Milli-Q water three times. After SLB formation, care was taken to keep the surface submerged in water and without bubbles.

### Analytical Techniques

^**1**^H **NMR and**^**13**^**C NMR** spectra were recorded on a Varian Mercury Vx 400 MHz (400 MHz for ^1^H NMR and 100 MHz for ^13^C NMR). Proton chemical shifts are reported in ppm (δ) downfield from trimethylsilane (TMS) using the resonance frequency of the deuterated solvent as the internal standard. Peak multiplicity abbreviated as s: singlet; d: doublet, q: quartet; p: pentet; m: multiplet; dd: double doublet; dt: double triplet; dq: double quartet. Carbon chemical shifts are reported in ppm (δ) downfield from TMS using the resonance frequency of the deuterated solvent as the internal standard. For ^**19**^**F NMR**, chemical shifts were measured with a recycle delay of 10 s and are reported in ppm (δ) downfield from CFCl_3_ as the internal standard.

**Matrix-assisted laser absorption/ionization mass time-of-flight mass spectrometry (MALDI-TOF-MS)** spectra were obtained on a Bruker Autoflex Speed. α-Cyano-4-hydroxycinnamic acid (CHCA) and *trans*-2-[3-(4-*tert*-butylphenyl)-2-methyl-2-propenylidene]malononitrile (DCBT) were used as matrices. All samples were dissolved in tetrahydrofuran.

**Liquid chromatography–mass spectrometry (LC-MS)** was performed on a system consisting of the following components: a Shimadzu SCL-10A VP system controller with Shimadzu LC-10AD VP liquid chromatography pumps (with an Alltima C18 3 μm (50 × 2.1 mm) reversed-phase column and gradients of water–acetonitrile supplemented with 0.1% formic acid), a Shimadzu DGU-20A3 Prominence degasser, a Thermo Finnigan Surveyor autosampler, a Thermo Finnigan Surveyor PDA detector, and a Thermo Scientific LCQ Fleet. Gradients were run from 5% MeCN to 100% MeCN over a 15 min period.

**Ultraviolet–visible (UV–vis)** absorbance spectra were recorded on a Jasco V-650 UV–vis spectrometer or a Jasco V-750 UV–vis spectrometer with a Jasco ETCT-762 temperature controller. Measurements were performed using Quartz cuvettes with a path length of 1 mm (500 μM samples) or 5 cm (5 μM samples). First, a baseline of the corresponding solvent was measured. All measurements were performed with a bandwidth of 1.0 nm, a scan speed of 100 nm/min, and a data interval of 0.1 nm, spanning the UV–vis range from 190 to 500 nm. All spectra were averaged over three measurements.

**Static Light Scattering (SLS)** measurements were performed on an ALV ALVCGS-3 Compact Goniometer equipped with an ALV5000 digital correlator and a HeNe laser operating at 532 nm. Scattering intensity was detected over the angular range of 30–150 degrees with steps of 5 degrees, with 10 runs of 10 s per angle. BTA samples were prepared at a concentration of 500 μM and were measured in light scattering tubes with an outer diameter of 1 cm. As a reference, samples of only the corresponding solvent and only toluene were measured. Water was filtered with a 0.2 μm syringe filter (Supor membrane, PALL Corporation), and toluene was filtered with a 0.2 μm syringe filter (PTFE membrane, Whatman). The measurements were analyzed with AfterALV (1.0d, Dullware) to remove data showing obvious scattering from dust. The Rayleigh ratio as a function of the angle was computed using the equation below with toluene as a reference:
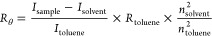
where *I*_sample_ is the count rate of the sample solution, *I*_solvent_ is the count rate for the solvent (water), and *I*_toluene_ is the count rate for toluene. *R*_toluene_ is the known Rayleigh ratio of toluene (2.1 × 10^–2^ m^–1^ at 532 nm), *n*_solvent_ is the refractive index of the solvent (1.333 for water), and *n*_toluene_ is the refractive index of toluene (1.497).

**Hydrogen/deuterium exchange mass spectrometry (HDX-MS)** measurements were carried out using a Xevo G2 QTof mass spectrometer (Waters) with a capillary voltage of 2.7 kV, a sampling cone voltage of 20 V, and an extraction cone voltage of 4.0 V. The source temperature was set at 100 °C, the desolvation temperature at 400 °C, the cone gas flow at 10 L h^–1^, and the desolvation gas flow at 100 L h^–1^. The sample solutions subjected to HDX were introduced into the mass spectrometer using a Harvard syringe pump (11 Plus, Harvard Apparatus) at a flow rate of 50 μL min^–1^. Previously prepared BTA samples of 500 μM in MQ water were diluted 100 times with D_2_O (including 0.5 mM sodium acetate to facilitate detection), resulting in a final concentration of 5 μM. MS spectra of supramolecular assemblies in water were recorded at several time points after dilution. The intensity of the peaks was used for the calculations as described in Section S3.

**Cryogenic transmission electron microscopy (cryo-TEM)** was performed on samples with a concentration of 500 μM of BTAs in water. Vitrified films were prepared in a “Vitrobot” instrument (FEI Vitrobot Mark IV, FEI Company) at 22 °C and at a relative humidity of 100%. In the preparation chamber of the “Vitrobot”, 3 μL samples were applied on Quantifoil grids (R 2/2, Quantifoil Micro Tools GmbH) or Lacey grids (LC200-Cu, Electron Microscopy Sciences), which were surface-plasma-treated just prior to use (Cressington 208 carbon coater operating at 5 mA for 40 s). Excess sample was removed by blotting using filter paper for 3 s with a blotting force of −1, and the thin film thus formed was plunged (acceleration of about 3 g) into liquid ethane just above its freezing point. Vitrified films were transferred into the vacuum of a CryoTITAN equipped with a field emission gun that was operated at 300 kV, a postcolumn Gatan energy filter, and a 2048 × 2048 Gatan CCD camera. Vitrified films were observed in the CryoTITAN microscope at temperatures below −170 °C. Micrographs were taken under low-dose conditions, starting at a magnification of 6500 with a defocus setting of −40 μm, and at a magnification of 24 000 with a defocus setting of −10 μm.

**Total internal reflection fluorescence (TIRF) microscopy** images were acquired with a Nikon N-STORM system. Cy5 was excited using a 647 nm laser, and AF488 was excited using a 488 nm laser. TIRF images were acquired at 5% (488 channel) or 8% (647 channel) laser power and 100 ms exposure time. Fluorescence was collected by means of a Nikon ×100, 1.4 NA oil immersion objective and passed through a quad-band-pass dichroic filter (97335 Nikon). Images were recorded with an EMCCD camera (iXon3, Andor, pixel size 0.16 μm).

## Results and Discussion

### Molecular Design and Synthesis

The design of the functional monomers was based on the well-known water-soluble **BTA-(OH)**_**3**_ (Figure 1A). Benzoxaborole (Ba)^33^ covalently attached to the BTA motif served as a surface-anchoring motif, due to its affinity for sialic acid at physiological and slightly acidic pH (**BTA-Ba**).^36−38^ A cyanine dye (Cy5) was attached for fiber visualization (**BTA-Cy5**).^26^ Two new BTA monomers for antibody binding were synthesized by linking the antibody-binding motif DNP to the periphery of **BTA-(OH)**_**3**_. To investigate the effect of the monomeric design on antibody binding, the functional monomers bore either one (**BTA-DNP**_**1**_) or three (**BTA-DNP**_**3**_) DNP groups (Figure 1A). First, the scope and limitations of the supramolecular copolymerization of antibody-binding monomers **BTA-DNP**_**1**_ and **BTA-DNP**_**3**_ with **BTA-(OH)**_**3**_ were studied. In addition, both Ba- and Cy5-dye were attached to the same BTA monomer to afford the surface-anchoring monomer **BTA-Ba-Cy5** for simultaneous visualization and binding. Following previous work to improve the stability and accessibility of functional groups, the antibody-recruiting dinitrophenyl motif (DNP) was attached via an additional linker.^33^ A supported lipid bilayer (SLB) was used as a cellular membrane mimic (Figure 1B). SLBs are known to have antifouling properties, as well as two-dimensional fluidity, making them suitable to study interactions on a cell-like surface.^39^ The SLB was functionalized with sialic acid via the incorporation of ganglioside GD3, a naturally occurring ganglioside presenting two sialic acid residues.^40^

**Scheme 1:**
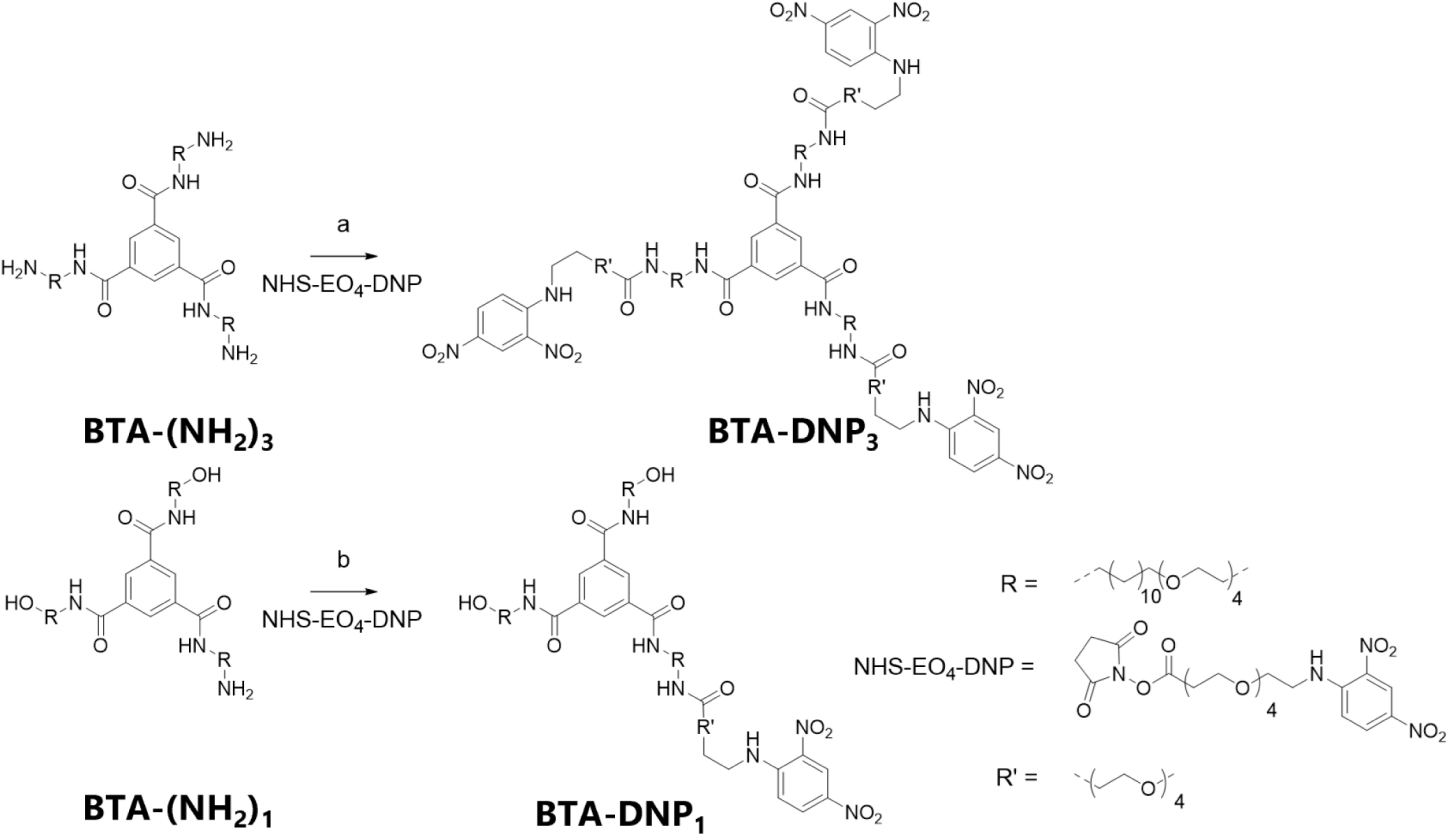
Synthetic Route toward **BTA-DNP**_**3**_ and **BTA-DNP**_1_ Reaction conditions: (a) NHS-EO4-DNP, TEA, DCM, 42% and (b) NHS-EO4-DNP, TEA, DCM, 19%.

The DNP-functionalized monomers were obtained via amine-modified BTA intermediates (**BTA-(NH**_**2**_**)**_**3**_ or **BTA-(NH**_**2**_**)**_1_), which reacted with the commercially available NHS ester of the DNP ethylene oxide linker (Scheme 1). **BTA-Ba-Cy5** was obtained from a previously described BTA precursor comprising an amine, an azide, and an alcohol group.^28^ The azide and amine were orthogonally functionalized with the Ba unit and the Cy5 dye. **BTA-Ba** and **BTA-Cy5** were obtained as reported previously.^26,33^ The purity and the assignment of the BTA structures were confirmed by ^1^H NMR, ^13^C NMR, matrix-assisted laser desorption ionization-time-of-flight mass spectrometry (MALDI-TOF-MS), and liquid chromatography–mass spectrometry (LC-MS) (see Supporting Information for details).

### DNP Functionalized Monomers Can Be Copolymerized with BTA-(OH)_3_

**Figure 2:**
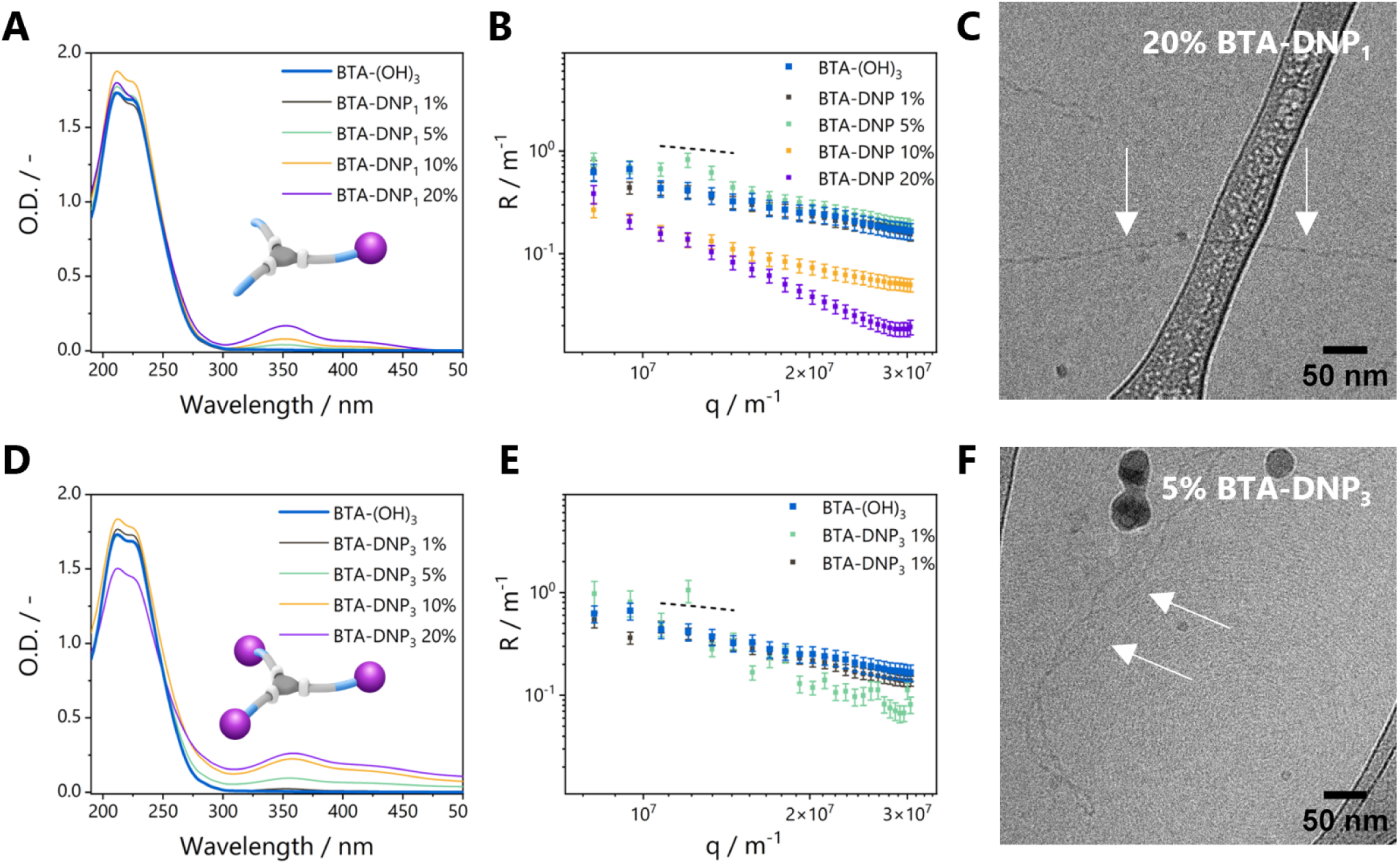
UV–vis spectra (A, D), SLS spectra (B, E), and cryo-TEM images (C, F) upon copolymerization of **BTA-(OH)**_**3**_ with different mol % of DNP-functionalized monomers (*c*_BTA, total_ = 500 μM, *T* = 20 °C). The dashed lines in the SLS spectra are guides to the eye denoting the −1 exponential decay. Fibers are indicated by the white arrows in the cryo-TEM images.

First, the copolymerization of the DNP-functionalized monomers **BTA-DNP**_**1**_ or **BTA-DNP**_**3**_ with **BTA-(OH)**_**3**_ was studied. Both **BTA-DNP**_**1**_ and **BTA-DNP**_**3**_ were insoluble in water, indicating that the introduction of the DNP moiety significantly impacted the hydrophobicity of the BTA-based monomers. To enable water solubility, supramolecular copolymers containing several mol percentages (0, 1, 5, 10, and 20 mol %) of **BTA-DNP**_**1**_ or **BTA-DNP**_**3**_ mixed with **BTA-(OH)**_**3**_ were prepared. **BTA-DNP**_**1**_**/BTA-(OH)**_**3**_ copolymers were investigated using UV–vis (Figure 2A). All showed absorption maxima at 211 and 226 nm similar to **BTA-(OH)**_**3**_ homopolymers, which is a first indication of the formation of polymers with structures similar to **BTA-(OH)**_**3**_ homopolymers. The additional absorption bands around 350 and 425 nm originate from the DNP moiety. Static light scattering (SLS) (Figure 2B) shows an angular dependency of the Rayleigh ratio typical for elongated structures (∝ q^–1^) for **BTA-(OH)**_**3**_ and low incorporation ratios of **BTA-DNP**_**1**_. Upon increasing the amount of **BTA-DNP**_**1**_ (10% and 20%), the intensity and slope of the scattering profile are altered. In case of **BTA-DNP**_**1**_ 10%, the similar slope implies the formation of elongated structures. For **BTA-DNP**_**1**_ 20%, the scattering profile also shows a drop in intensity as well as a change in slope, suggesting shortening of the length of the formed assemblies. Cryogenic transmission electron microscopy (cryo-TEM) images (Figure 2C) of the 20% copolymer show fibrillar structures with chain ends, corroborating the shortening of the fibers as seen with SLS for high incorporation ratios of **BTA-DNP**_**1**_.

Copolymerization of **BTA-DNP**_**3**_ with **BTA-(OH)**_**3**_ also showed absorption maxima at 211 and 226 nm and additional absorptions at 350 and 425 nm (Figure 2D). However, scattering is visible at higher wavelengths, especially at higher percentages of **BTA-DNP**_**3**_ (10% and 20%), indicative of inhomogeneous solutions caused by precipitates. These mixtures were therefore not further investigated. At the lowest incorporation percentages (1% and 5%), SLS (Figure 2E) showed a scattering profile resembling that of **BTA-(OH)**_**3**_ homopolymers. Cryo-TEM images of the 5% copolymer revealed fibrillar structures (Figures 2F and S17) similar to those of the 1% copolymer (Figure S17). This suggests that at low percentages of the incorporation of **BTA-DNP**_**3**_ monomers, supramolecular polymers can still be formed.

**Figure 3:**
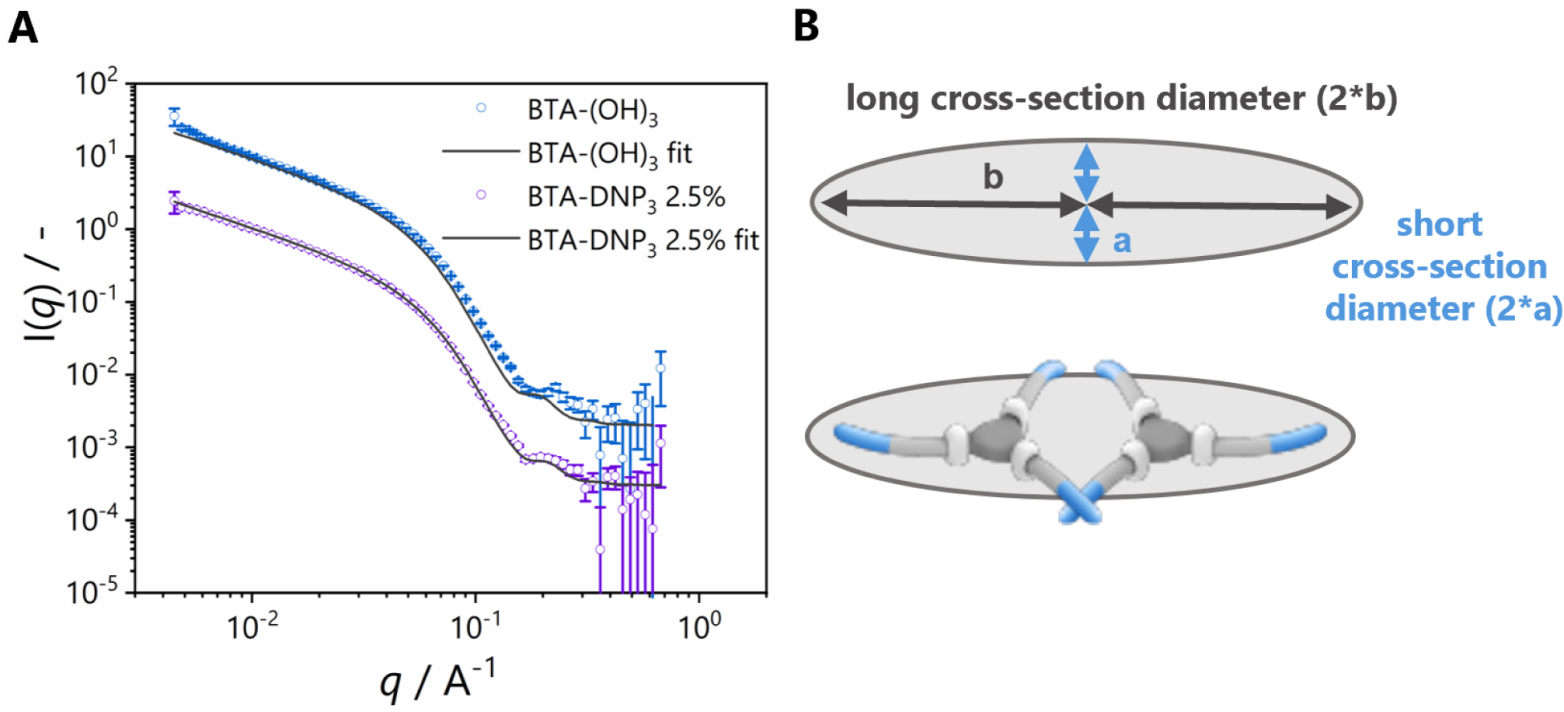
(A) SANS scattering profiles and corresponding elliptical cylinder fits in D_2_O. Data are shifted vertically for clarity (*c* = 4.5 mM). (B) Schematic representation of the cross-sectional diameter of the elliptical cylinder.

To further understand the effect of copolymerizing DNP-functionalized monomers, small-angle neutron scattering (SANS) experiments were performed (Figure 3A) on **BTA-(OH)**_**3**_ homopolymers and **BTA-(OH)**_**3**_ copolymerized with 2.5% **BTA-DNP**_**3**_. The scattering profile at low *q* did not form a plateau, indicating the formation of long objects. Moreover, the shape of the scattering profiles for both **BTA-(OH)**_**3**_ and the **BTA-DNP**_**3**_**:BTA-(OH)**_**3**_ copolymer was similar (see Figure S18 for an overlay of the scattering curves). Power law analysis at low *q* revealed slopes of −1 for both assemblies, indicating cylindrical structures in solution. **BTA-(OH)**_**3**_ is best described by a cylinder with an elliptical cross-section to capture the small peak at high *q*. A schematic representation of the cross-section is shown in Figure 3B. The axis ratio of 1.87 between the short and long cross-sectional diameters of the cylinder matches a cross-section composed of 2 BTA monomers (Figure 3B), which agrees well with the formation of a double-helical structure previously elucidated for **BTA-(OH)**_**3**_ homopolymers.^27^ The parameters obtained from the elliptical cylinder fits are summarized in Table S1. Upon incorporation of **BTA-DNP**_**3**_, the cylindrical diameter and axis ratio were not affected, indicating that the copolymerization of **BTA-(OH)**_**3**_ with 2.5% **BTA-DNP**_**3**_ retained the morphology and the size of the formed copolymers.

Summarizing the results above indicates the successful copolymerization of **BTA-DNP**_**1**_ and **BTA-DNP**_**3**_ with **BTA-(OH)**_3_, yielding DNP-functionalized supramolecular polymers with a morphology similar to homopolymers of **BTA-(OH)**_**3**_. However, the maximum incorporation percentage depends on the valency of the BTA-DNP (i.e., 1 or 3).

### Back-Folding of DNP Motifs Studied by Molecular Modeling

**Figure 4:**
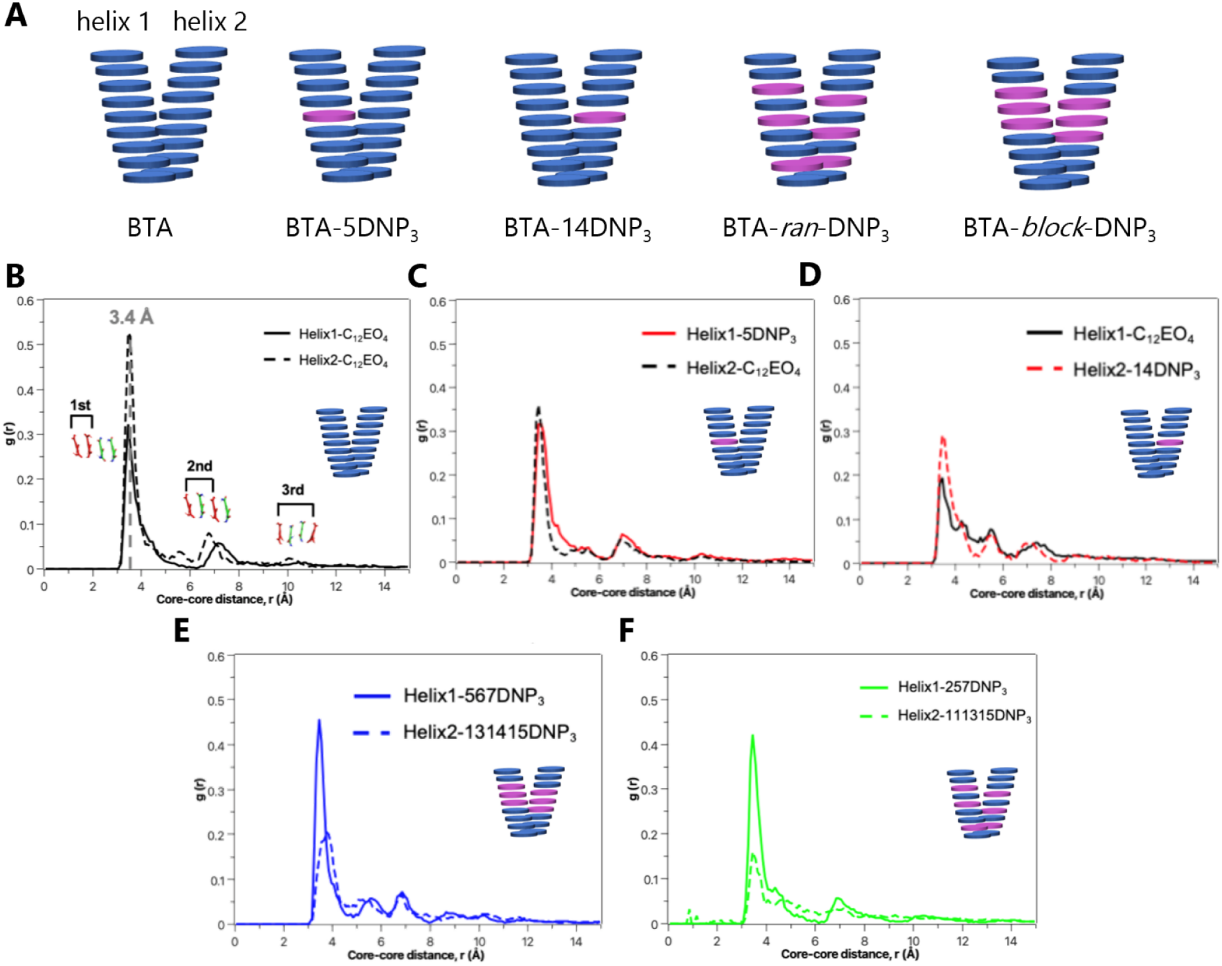
(A) Schematic representation of the double helices containing 0, 1, and 6 **BTA-DNP**_**3**_ monomers with different monomer sequences. **BTA-DNP**_**3**_ monomers are depicted in purple. (B–F) Radial distribution functions *g*(r) of the BTA cores along the two helices in (B) **BTA-(OH)**_**3**_ homopolymer, (C) BTA-5DNP_3_ copolymer, (D) BTA-14DNP_3_ copolymer, (E) BTA-*ran*-DNP_3_ copolymer, and (F) BTA-*block*-DNP_3_ copolymer.

To gain a better understanding of the self-assembly mode and fluctuations of the monomers within the copolymers, all-atom molecular dynamics (MD) simulations were performed. The MD simulations were performed on copolymers composed of **BTA-(OH)**_**3**_ and **BTA-DNP**_**3**_ since **BTA-DNP**_**3**_ contains the highest functionality compared to **BTA-DNP**_**1**_. The copolymers were studied in an implicit water solvent, with different mixing ratios and monomer sequences, as well as taking into account the double-helical morphology of BTA-based systems. The assembly and stability behavior of the copolymers were compared to MD simulations of a **BTA-(OH)**_**3**_ homopolymer in a double-helical morphology. Five pre-equilibrated double fibers of each 9 monomers in length (i.e., 18 monomers in total) containing 0, 1, or 6 **BTA-DNP**_**3**_ monomers within **BTA-(OH)**_**3**_ hosts were constructed (BTA, BTA-5DNP_3_, BTA-14DNP_3_, BTA-*ran*-DNP_3_, and BTA-*block*-DNP_3_) (Figure 4A). Their geometries were optimized and used as starting structures for MD simulations on a 500 ns timescale (see computational details in the Supporting Information). In the early timescales of the MD simulations, the supramolecular fibers underwent folding in water to reduce the hydrophobic area exposed to the solvent. Due to this hydrophobically driven folding and bending of the fibers, the BTA cores were not perfectly aligned along a 1D stacking direction, as demonstrated in earlier studies.^41,42^

A useful indicator of the level of stacking order within the fibers is derived from the radial distribution function *g*(r) between the BTA cores, indicating the probability of finding neighboring BTA units as a function of their distance.^30,41−43^ The *g*(r) of the BTA cores in each helix for the **BTA-(OH)**_**3**_**:BTA-DNP**_**3**_ copolymers was compared to the **BTA-(OH)**_**3**_ homopolymer (Figure 4B–F). The *g*(r) plots show peaks at the characteristic BTA-stacking distances (first peak: first closest neighbor, second peak: second neighbor, third peak: third neighbor, etc.). In general, higher *g*(r) peaks indicate more ordered, stable, and persistent stacking of the core in the fibers. The *g*(r) plot of the **BTA-(OH)**_**3**_ homopolymer contains three characteristic peaks at distances of 3.4, 6.8, and 10.2 Å, which are typical of ordered stacking (Figure 4B), whereas the *g*(r) plots of the **BTA-(OH)**_**3**_**:BTA-DNP**_**3**_ copolymers show clear first, second, and third peaks for one helix but lack clear second and third peaks for the second helix, indicating that the stacking order is only present up to the first closest neighbors (Figure 4C–F). Thus, in the **BTA-(OH)**_**3**_**:BTA-DNP**_**3**_ copolymers, the core–core stacking seemed to be more ordered in one helix with respect to the second helix. Compared to **BTA-(OH)**_**3**_ homopolymer, the overall stacking was less ordered.

**Figure 5:**
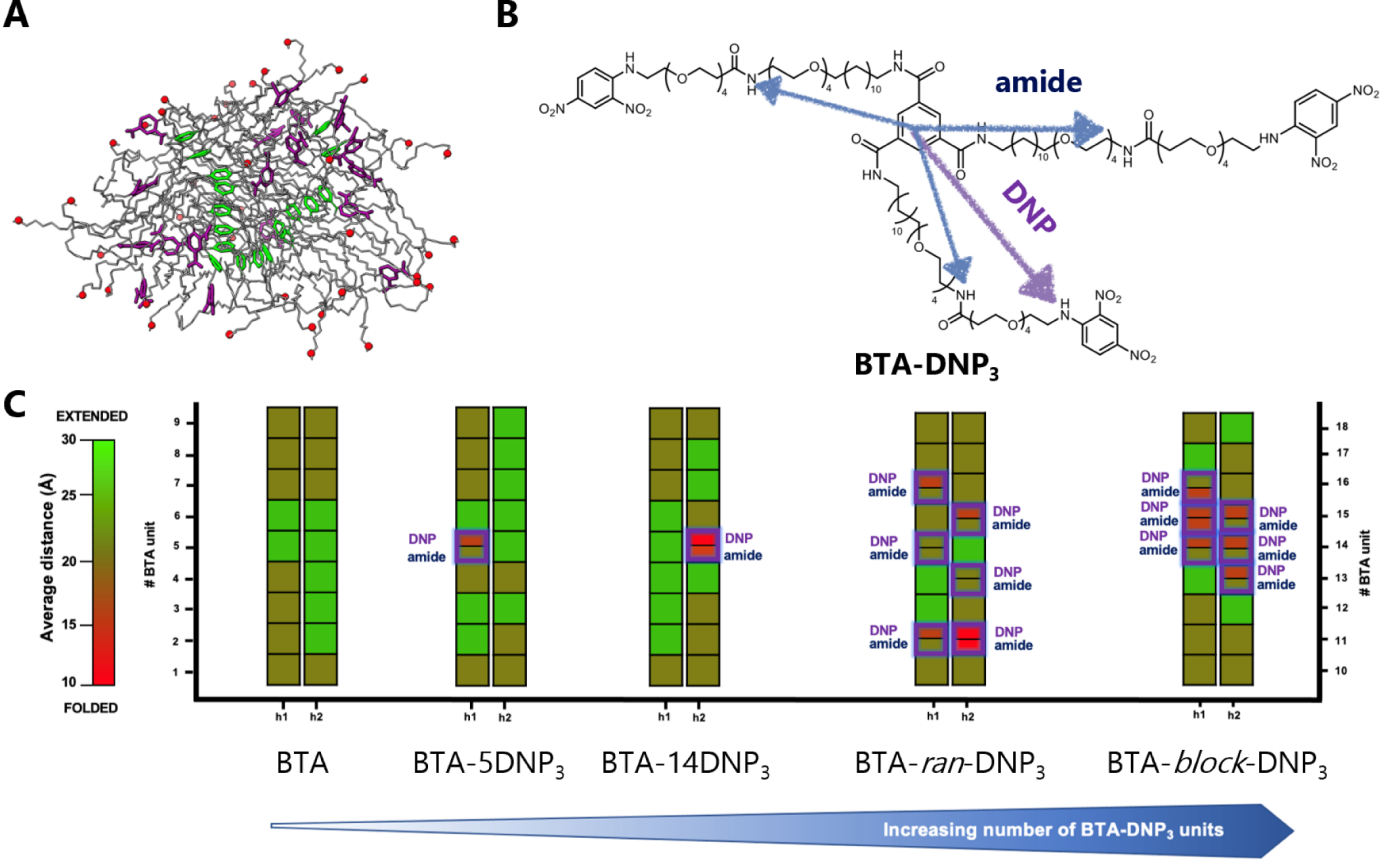
(A) Final snapshot of MD simulations of the BTA-*ran*-DNP_3_ copolymer. The carbon atoms of the BTA cores are depicted in green, the side chains in gray, the DNP motifs in purple, and the oxygen atoms of the −OH of the side chains are depicted in red spheres. (B) Scheme showing the selected atoms to compute the degree of extension of the side chains. (C) The degree of extension of the side chains of each BTA unit as the average distances between the BTA cores and the selected atoms of the side chains. Each square corresponds to one BTA unit in the two helices (h1 and h2) of the fibers (starting from the first BTA unit in each helix). The side chains of the BTA units that are the most folded are represented by low distances (red squares) and marked with purple borders.

The folding of the side chains was investigated to gain insight into the accessibility of the DNP motifs for antibody binding. To this end, the degree of extension of the side chains was assessed by evaluating the average distances between the cores of each BTA unit and the −OH of the side chains (for **BTA-(OH)**_**3**_ monomers), or between the cores of each BTA unit and the −NH of the side chains at the start of the linker (amide) or the −NH of the DNP (for **BTA-DNP**_**3**_ monomers) (Figure 5B). The degree of extension of the side chains of each BTA unit in the supramolecular fibers was evaluated by comparing the color-coded maps for **BTA-(OH)**_**3**_ with **BTA-(OH)**_**3**_**:BTA-DNP**_**3**_ copolymers (Figure 5C). For **BTA-(OH)**_**3**_, the side chains of the BTA units are mostly extended, with the −OH group pointing outward and exposed to the solvent, as revealed by the large distances (>25 Å). In contrast, the color-coded maps of BTA-5DNP_3_ and BTA-14DNP_3_ copolymers showed that, on average, the degree of extension of the side chains of the **BTA-DNP**_**3**_ units is below 20 Å. This indicates that the DNP-containing side chains are back-folded into the fibers, with the DNP moieties relatively close to the BTA cores. The copolymers with the highest numbers of **BTA-DNP**_**3**_ monomers (BTA-*ran*-DNP_3_ and BTA-*block*-DNP_3_) showed a higher degree of folded regions, which was more pronounced for BTA-*block*-DNP_3_ (higher number of red regions in Figure 5C). Altogether, these results indicate that DNP motifs point mostly inward while the −OH tends to point outward exposed to the solvent (Figure 5A). In addition, no persistent stacking interactions were detected between the DNP moieties, indicating that the folding of the DNP side groups is mostly driven by hydrophobic effects. This back-folding could have an effect on antibody recruitment due to the limited availability of the DNP unit (see below).

### Multicomponent Copolymers of BTA-Ba, BTA-DNP_1/3_, and BTA-(OH)_3_

**Figure 6:**
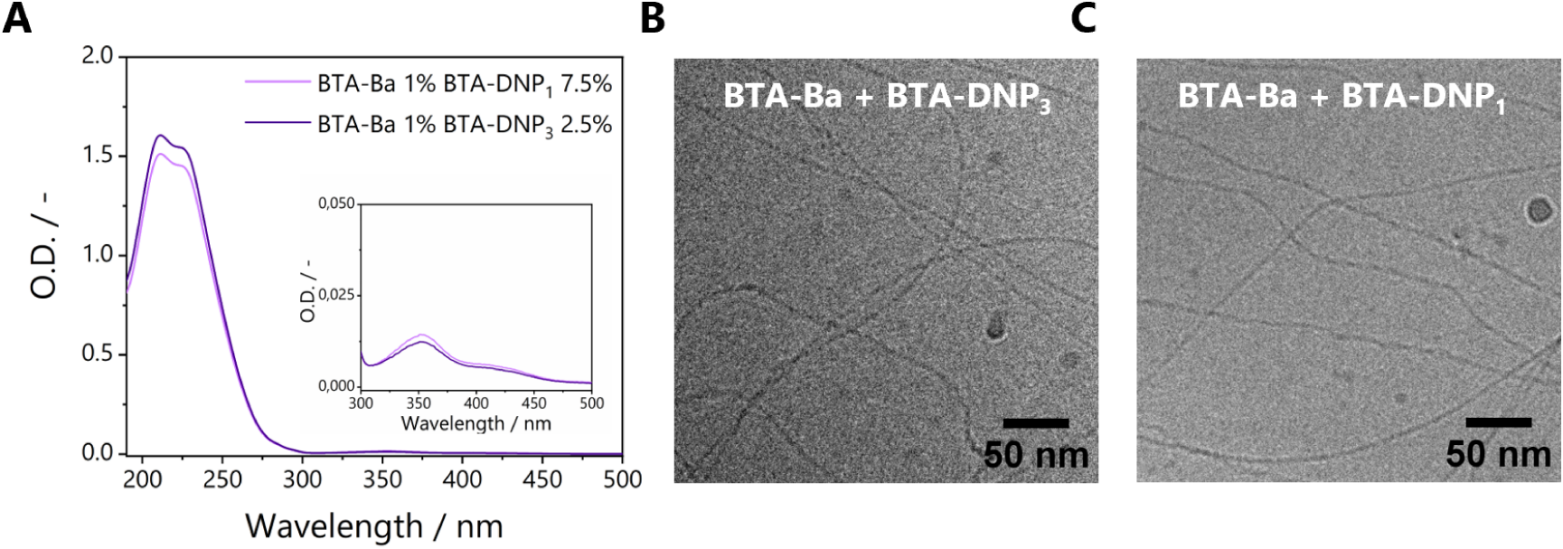
UV–vis (A) and cryo-TEM images (B, C) and UV–vis upon copolymerization of **BTA-(OH)**_**3**_ with **BTA-Ba** and **BTA-DNP**_**1**_ or **BTA-DNP**_**3**_ (*c*_BTA_ = 500 μM, *T* = 20 °C).

The results described above indicate the successful formation of copolymers comprising **BTA-(OH)**_**3**_ and **BTA-DNP**_**1**_ or **BTA-(OH)**_**3**_ and **BTA-DNP**_**3**_ (up to 5 mol %), with structural characteristics similar to **BTA-(OH)**_**3**_ homopolymers, and hence negligible disturbance in the incorporation of functional entities. As a next step, a multicomponent system was investigated by copolymerizing **BTA-(OH)**_**3**_, **BTA-Ba**, and either **BTA-DNP**_**1**_ or **BTA-DNP**_**3**_. As only a small percentage of **BTA-Ba** is required for surface anchoring, only 1 mol % of this monomer was included,^33^ while **BTA-DNP**_**3**_ was included at a 2.5 mol % incorporation to prevent the formation of precipitates or shortening of the assemblies. **BTA-DNP**_**1**_ was included at 7.5 mol % to keep the DNP concentration constant. UV–vis (Figure 6A) showed the typical absorption maxima at 211 and 226 nm, and small DNP-derived peaks at 350 and 425 nm. At higher wavelengths, no scattering was visible, indicating the homogeneity of the prepared samples. Cryo-TEM images confirmed the presence of long fibrillar structures without chain ends for both multicomponent copolymers (Figure 6B,C).

### Effect of DNP Motif on Monomer Dynamics

**Figure 7:**
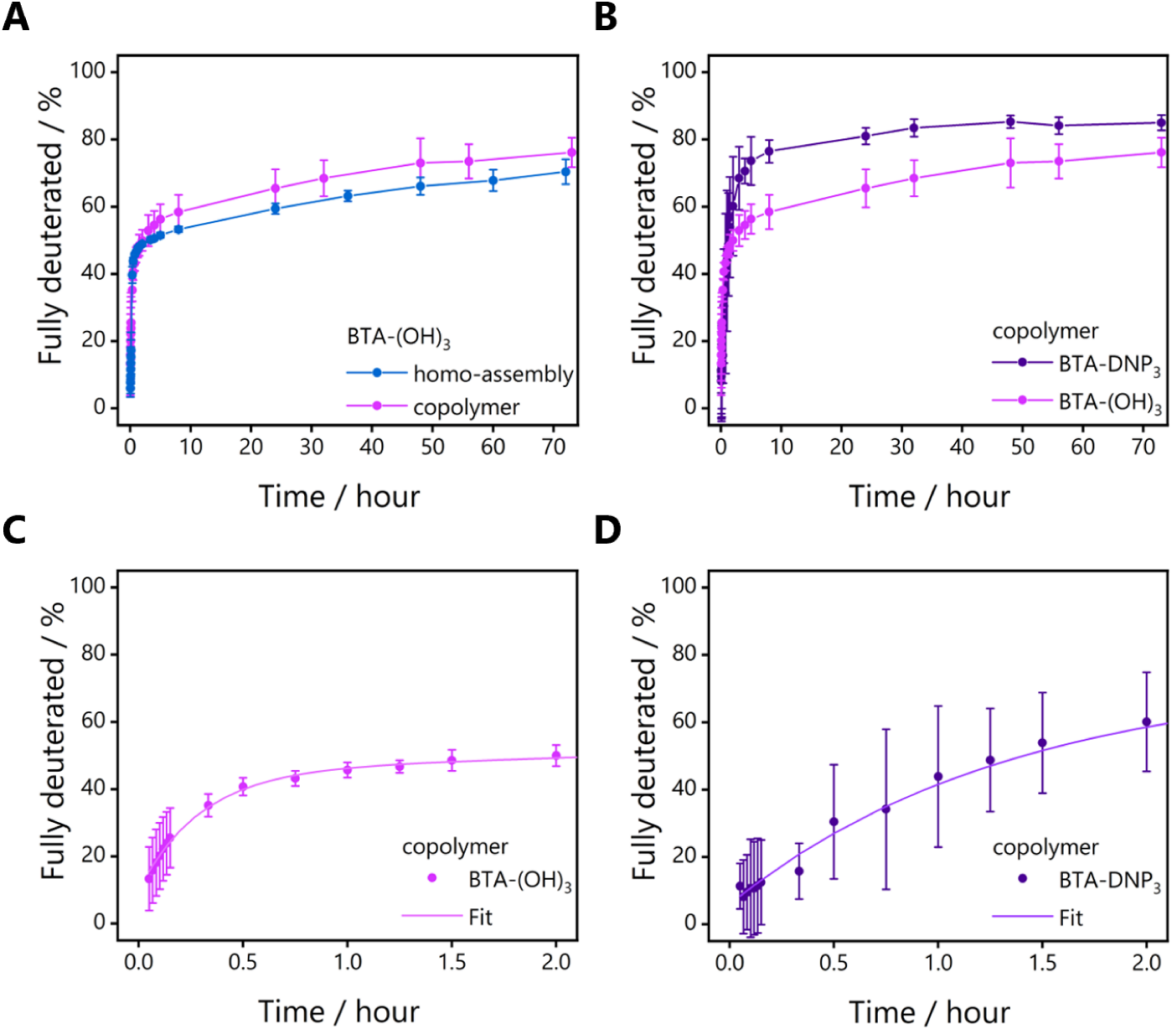
HDX-MS curves of after 100 times dilution of an aqueous sample into D_2_O (initial *c* = 500 μM, after dilution *c* = 5 μM, *T* = room temperature) of **BTA-(OH)**_**3**_ (A, C) and **BTA-DNP**_**3**_ (B, D) monomers within a **BTA-(OH)**_**3**_ homoassembly (A) and within a **BTA-DNP**_**3**_:**BTA-Ba**:**BTA-(OH)**_**3**_ (2.5:1:96.5) copolymer (B–D). The graphs show the amount of fully deuterated monomers as a function of time. The error bars represent the standard deviation calculated from three separate experiments. Lines are added to guide the eye (A, B) or represent a biexponential growth function (C, D).

To gain insight into the incorporation of functionalized monomers into the supramolecular polymers, HDX-MS was employed, previously developed in our group to probe monomer exchange dynamics.^25,44^ Herein, we focused on a copolymer composed of **BTA-DNP**_**3**_**:BTA-Ba:BTA-(OH)**_**3**_ (2.5:1:96.5) since **BTA-DNP**_**3**_ differs the most in chemical structure from **BTA-(OH)**_**3**_ in comparison to **BTA-DNP**_**1**_. The BTAs have either six (**BTA-(OH)**_**3**_) or nine (**BTA-DNP**_**3**_) exchangeable hydrogen atoms (NH and/or OH), and the exchange of these hydrogen atoms to deuterium upon dilution into D_2_O can be followed with MS. Previous studies showed that the outer hydroxyl or amide groups exchange immediately upon dilution, while the inner amides predominantly exchange upon monomer migration into the surrounding D_2_O as they are shielded from D_2_O in the hydrophobic pocket of the supramolecular stack.^44^ Thus, the H/D exchange can be related to monomer dynamicity. In addition, the H/D exchange can also give insights into the internal order of the supramolecular stack by investigating the intermediate deuterated species. D_2_O can penetrate less ordered parts of the polymer, exchanging the labile hydrogen atoms one at a time, which gives rise to intermediate species (BTA4D and BTA5D for **BTA-(OH)**_**3**_ and BTA7D and BTA8D for **BTA-DNP**_**3**_, respectively). To study the H/D exchange, 500 μM BTA solutions were prepared in MQ water and diluted 100 times into D_2_O. Upon dilution, the nature of the aggregates did not change (Figure S19). To compare the different BTA-based monomers, the percentage of fully deuterated monomers (6D or 9D, see Supporting Information for details of the calculations of the percentages of deuterated species) was plotted as a function of time, as this reflects the monomer dynamicity (Figure 7A,B): the faster full deuteration is reached, the more dynamic the system is. The intensity of the MS peaks of the 1 mol % **BTA-Ba** was unfortunately too low to be reliably tracked with HDX-MS, while the intensity of 2.5 mol % **BTA-DNP**_**3**_ was sufficiently high to reliably interpret.

First, the progression of 6D species of **BTA-(OH)**_**3**_ present in either the homopolymer or the copolymer **BTA-DNP**_**3**_**:BTA-Ba:BTA-(OH)**_**3**_ (2.5:1:96.5) was compared (Figure 7A). Figure 7A shows that the exchange profile of **BTA-(OH)**_**3**_ is slightly altered in the copolymer compared to the homopolymer, which indicates the successful incorporation of **BTA-DNP**_**3**_ into the copolymer; a lack of incorporation of **BTA-DNP**_**3**_ would result in an identical exchange profile of **BTA-(OH)**_**3**_ as compared to the homopolymer. Figure 7B shows the exchange profile of either **BTA-(OH)**_**3**_ or **BTA-DNP**_**3**_ monomers within the copolymer. Interestingly, **BTA-DNP**_**3**_ showed a higher percentage of fully deuterated species (80% after 70 h) compared to **BTA-(OH)**_**3**_ (60% after 70 h). Analysis of the intermediate deuterated species for both **BTA-(OH)**_**3**_ (Figure S20A) and **BTA-DNP**_**3**_ (Figure S20B) in the first 2 h of the exchange experiment revealed a higher percentage of intermediate deuterated species (BTA7D, BTA8D) for **BTA-DNP**_**3**_ compared to **BTA-(OH)**_**3**_ (BTA4D, BTA5D). Both the higher deuteration degree as well as higher amounts of nonfully deuterated species are observed for **BTA-DNP**_**3**_ in the copolymers. This has been attributed to a higher probability of water penetration into the hydrophobic regions of the copolymer and a higher likelihood of monomer exchange.^44^ As a result, we can conclude that **BTA-DNP**_**3**_ resides within less ordered parts of the supramolecular copolymer. Comparison of the data for **BTA-(OH)**_**3**_ in the homo- versus the copolymer showed also a slight decrease in order for the **BTA-(OH)**_**3**_ monomers, most probably due to the units that are closest to the **BTA-DNP**_**3**_ monomers (Figure 7A).

To gain further insight into the exchange dynamics of the monomers within the copolymer, the percentage of fully deuterated monomers was fitted with a biexponential growth function using a fast (*k*_fast_) and slow (*k*_slow_) rate constant as well as a relative contribution of both processes (Figure 7C,D and Table 1, details in the Supporting Information). Whereas the *k*_slow_ shows a similar value for both BTAs, the *k*_fast_ is about 3.5 times slower for **BTA-DNP**_**3**_ as compared to **BTA-(OH)**_**3**_ (Table 1), which was also noticed when analyzing the exchange profile at short timescales (Figure 7C,D). In addition, Table 1 shows that the contribution of *k*_fast_ to the exchange process (65%) for **BTA-DNP**_**3**_ is significantly higher than the contribution of *k*_fast_ for **BTA-(OH)**_**3**_ (35%). As the fast exchange process is related to initial exchange due to solvent penetration,^44^ this strongly suggests that the exchange of **BTA-DNP**_**3**_ is mostly driven by solvent penetration into less ordered parts of the polymer. The lower *k*_fast_ for **BTA-DNP**_**3**_ suggests that these monomers are buried in hydrophobic parts of the polymer, where solvent penetration is slowed down. Finally, the higher percentage of fully deuterated species for **BTA-DNP**_**3**_ compared to **BTA-(OH)**_**3**_ found in the HDX-MS measurements corroborates with the results described above, in which the back-folding of the DNP unit within the copolymers resulted in a reduction in order within the double-helical fibers as revealed by MD simulations.

**Table 1:** Rate Constants, *k*, and Contributions of the H/D Exchange Processes within the Copolymer Obtained after Fitting the Percentage of Molecules with All Hydrogen Atoms Exchanged for Deuterium Atoms with a Biexponential Growth Function

	Rate constants (h^–1^)		Contributions (%)	
	*k*_fast_	*k*_slow_	Fast	Slow
**BTA-(OH)**_**3**_	3.45	0.08	35.5	28.6
**BTA-DNP**_**3**_	0.76	0.05	65.5	14.2

### Surface Anchoring and Antibody Recruitment of BTA-Based Copolymers

**Figure 8:**
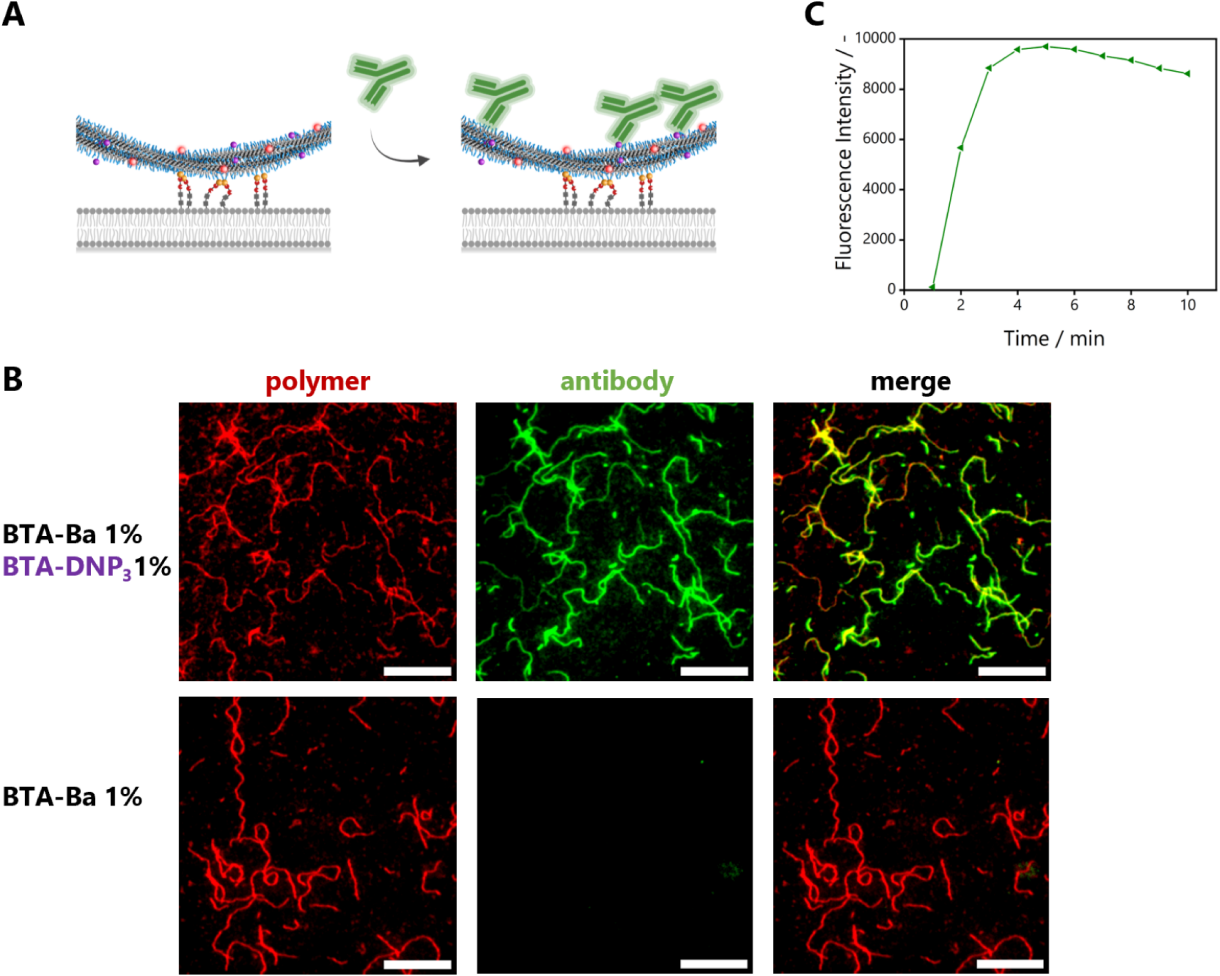
Antibody binding to anchored BTA fibers on an SLB. (A) Schematic representation of antibody binding toward anchored BTA fibers. (B) TIRF images of antibody binding, BTA labeled with Cy5 in the red polymer channel, antibody anti-DNP labeled with AF488 in the green antibody channel (*c*_BTA, total_ = 1 μM (**BTA-Ba** 1 mol %, **BTA-Cy5** 5 mol %, and **BTA-DNP**_3_ 0 or 1 mol %); *c*_anti-DNP_ = 0.002 mg/mL; scale bar represents 10 μm). (C) Fluorescence intensity of the AF488 channel upon antibody addition, obtained via analysis of masked images of BTA fibers (see Supporting Information for details).

Since MD simulations indicated back-folding of the DNP moieties, the accessibility of the DNP moieties toward interaction with anti-DNP antibodies might be affected as well as the interaction of the functionalized supramolecular polymers with cell-mimicking surfaces via Ba. To investigate this, a supported lipid bilayer (SLB) presenting sialic acid was used as a mimic of the phospholipid cell membrane. Supramolecular polymers were incubated on the sialic acid-functionalized SLB, followed by the incubation with AlexaFluor488-labeled anti-DNP antibody (AF488–anti-DNP) (Figure 8A). All copolymers were prepared comprising 1 mol % **BTA-Ba** for surface anchoring and 5 mol % **BTA-Cy5** for visualization of the BTA backbone using total internal reflection fluorescence (TIRF) microscopy and with varying amounts of **BTA-DNP**_**3**_. Upon incorporation of the **BTA-DNP**_**3**_ moiety within the polymers, the BTA fibers showed clear antibody binding in the AF488 fluorescence channel (Figure 8B, top middle). The fibers also retained their morphology upon interaction with anti-DNP antibodies. Thus, although the DNP moiety introduces disorder within the supramolecular polymers and is rather hydrophobic, it remains accessible for antibody binding.

Subsequently, the specificity of antibody binding was investigated as **BTA-(OH)**_**3**_ fibers have shown a small degree of nonspecific interactions previously, e.g., with naturally occurring proteins such as bovine serum albumin (BSA).^45^ No antibody binding was visible without the incorporation of **BTA-DNP**_**3**_ in the copolymers (Figure 8B, bottom middle), ruling out nonspecific interactions of BTA fibers with anti-DNP antibodies. To follow the progression of antibody binding events to BTA fibers, the fluorescence intensity of the AF488 channel upon the addition of anti-DNP to surface-anchored fibers was monitored over time (Figure 8C, details on image analysis in the Supporting Information). A rapid saturation of the signal occurred (∼4 min), which is consistent with the strong affinity reported for the DNP/anti-DNP interaction (with an equilibrium dissociation constant in the nanomolar range).^46^ These results show that the back-folding of the DNP units within BTA-based fibers does not negatively influence the recognition with the antibody.

### Influence of Monomer Design on Antibody Binding

**Figure 9:**
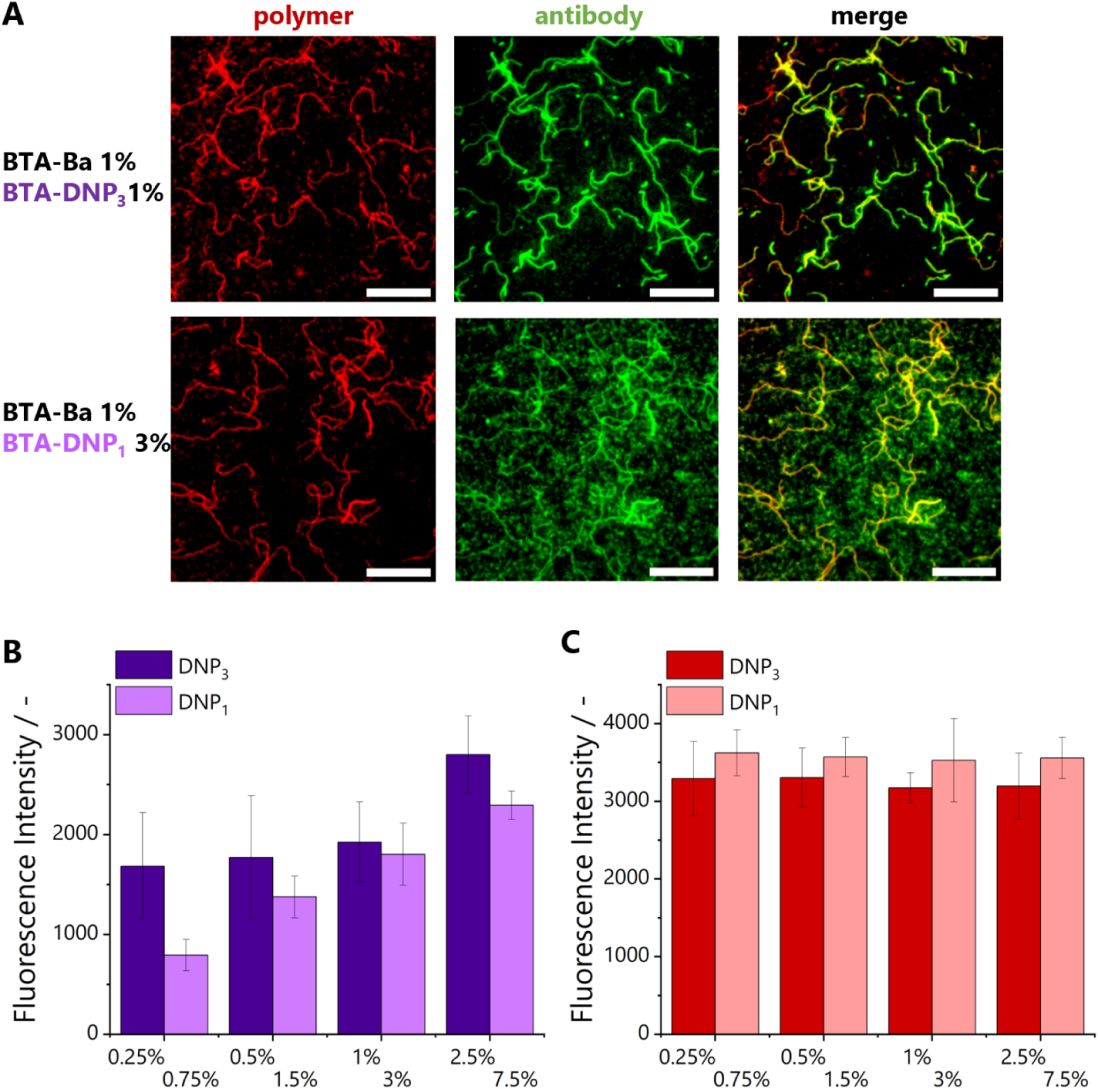
(A) TIRF images of antibody binding, BTA copolymers labeled with 5 mol % **BTA-Cy5** in the red polymer channel, antibody anti-DNP labeled with AF488 in the green antibody channel (*c*_BTA, total_ = 1 μM (**BTA-Ba** 1%, **BTA-Cy5** 5%, and varying amounts of **BTA-DNP**_**1/3**_ with respect to total BTA concentration); *c*_anti-DNP_ = 0.002 mg/mL; scale bar represents 10 μm). (B) Quantification of TIRF images of green channels. (C) Quantification of TIRF images of red channels (details on image analysis in the Supporting Information).

The effect of the presentation of DNP on antibody binding was investigated by comparing BTA copolymers comprising varying amounts of either **BTA-DNP**_**1**_ or **BTA-DNP**_**3**_, yet with similar molar amounts of DNP incorporated, i.e., three times as much **BTA-DNP**_**1**_ as compared to **BTA-DNP**_**3**_ (0.75–1.5–3–7.5 mol % DNP with regard to total BTA concentration). Representative images of copolymers bearing either **BTA-DNP**_**3**_ or **BTA-DNP**_**1**_ are shown in Figure 9A. Long fibers were visible in the red channel for both types of copolymers after anti-DNP incubation, demonstrating that copolymers with **BTA-DNP**_**1**_ are also available for binding with antibodies. Interestingly, for all incorporation percentages studied, the copolymers containing **BTA-DNP_3_** showed a more localized emission in the green channel, corresponding to binding to BTA fibers as indicated in the merged image. For copolymers containing **BTA-DNP**_1_, the emission in the green channel is more diffuse, suggesting that **BTA-DNP**_**1**_ is less incorporated into the copolymers compared to **BTA-DNP**_**3**._ In order to understand this phenomenon, the green AF488 fluorescence images were further analyzed to compare antibody binding between **BTA-DNP**_**3**_ and **BTA-DNP**_**1**_ by measuring the intensity in the antibody channel originating from the fibers relative to the background (Figure 9B). The same analysis was done for the red fluorescence intensity (Figure 9C, all details on image analysis are provided in the Supporting Information). The presentation of DNP on **BTA-DNP**_**3**_ slightly increased the antibody binding toward BTA fibers at all concentrations studied, possibly due to a higher local concentration of DNP for **BTA-DNP**_**3**_ monomers as compared to **BTA-DNP**_**1**_. In contrast, the adsorption of **BTA-DNP**_**3**_ or **BTA-DNP**_**1**_ fibers remained constant, regardless of the amount of functional monomer included. This indicates that the observed difference in green fluorescence intensity is not due to differences in primary adsorption of the BTA filaments but rather to a more efficient binding of antibodies to **BTA-DNP**_**3**_, owing to multivalency.

### Dynamicity of Anchored Copolymers

After confirming the specific interactions between DNP-functionalized supramolecular copolymers and anti-DNP antibodies, we followed the dynamic exchange of functional monomers of the supramolecular copolymers over time using TIRF microscopy. The intrinsic dynamic nature of supramolecular copolymers should allow for the dynamic reconfiguration of monomers to occur from one fiber to the other. More specifically, we evaluated whether DNP-functionalized monomers can exchange from nonbinding fibers to binding fibers anchored on a surface. To this end, we selected **BTA-DNP**_**3**_ functionalized fibers as these showed the highest antibody binding. To suppress additional exchange of dye monomers, the bifunctional **BTA-Ba-Cy5** was used for anchoring to allow simultaneous binding and visualization.

**Figure 10:**
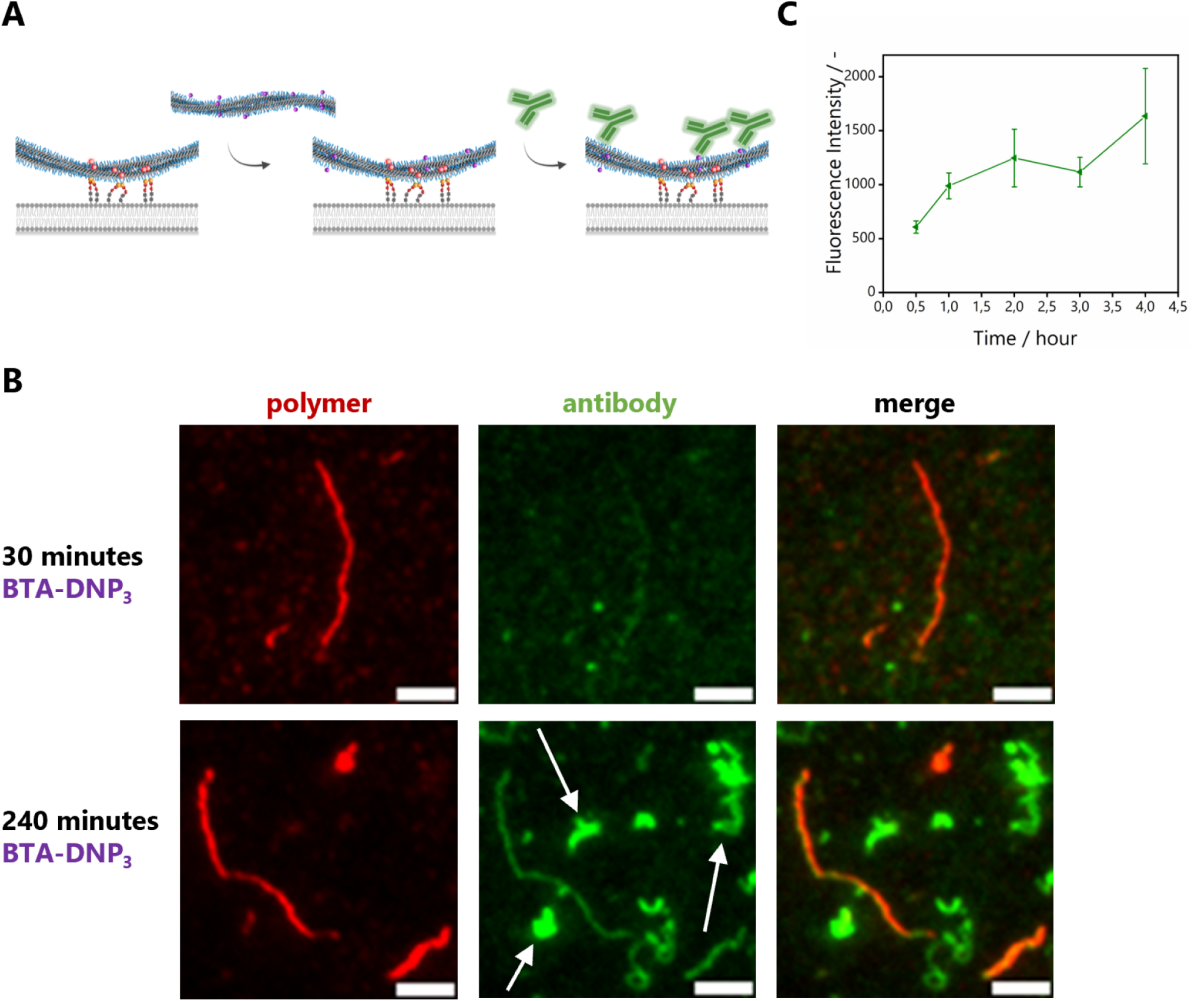
(A) Schematic representation of the dynamic exchange of **BTA-DNP**_**3**_ monomers from fibers in solution toward anchored fibers on an SLB. (B) TIRF images of BTA exchange experiments on an SLB, followed by antibody binding (*c*_BTA, SLB_ = 1 μM (**BTA-Ba-Cy5** 5 mol % with respect to total BTA concentration); *c*_BTA, solution_ = 0.1 μM (**BTA-DNP**_**3**_ 2.5 mol % with respect to total BTA concentration); and *c*_anti-DNP_ = 0.002 mg/mL; scale bar represents 5 μm). The arrows in the green channel indicate nonspecific surface binding of **BTA-DNP**_**3**_. (C) Intensity from the antibody channel upon incubation of anchored **BTA-(OH)**_**3**_ fibers with **BTA-DNP**_**3**_ over time. Only the fluorescence intensity originating from antibodies localized on red BTA fibers was taken into account (details on image analysis in the Supporting Information).

Starting from copolymers bearing **BTA-Ba-Cy5** anchored on a surface, free **BTA-DNP**_**3**_ functionalized copolymers were added and incubated for several time points. Subsequently, the fibers were incubated with anti-DNP antibody, which only binds to the anchored fibers if the exchange of **BTA-DNP**_**3**_ monomers has occurred. The results are visualized in Figure 10A. Interestingly, on short timescales (0–30 min), almost no increase in the green anti-DNP antibody channel is visible, indicating that little to no exchange of **BTA-DNP**_**3**_ to surface-anchored fibers occurred (Figure 10B, top). Gratifyingly, on longer timescales, the intensity in the green channel originating from the supramolecular red fibers increased (Figure 10C, details on image analysis in the Supporting Information), indicating a homogeneous exchange of **BTA-DNP**_**3**_ throughout the surface-anchored polymers (Figure 10B, bottom). However, it remains challenging to describe, at the molecular level, the mechanism of the different exchange processes taking place in this multicomponent system. Fully rationalizing how exactly the **BTA-DNP**_**3**_, present in fibers in solution, moves to fibers attached to the SLB surface is currently beyond our reach due to limitations of the currently available techniques.^33^

## Conclusions

The potential of BTA-based supramolecular polymers as multicomponent assemblies for antibody recruitment was studied with a new set of functional BTA monomers bearing either one or three DNP groups at their periphery. The introduction of DNP reduced the water compatibility of the monomers due to the hydrophobicity of DNP. Gratifyingly, upon copolymerization with **BTA-(OH)**_**3**_, supramolecular copolymers were realized with structural characteristics similar to **BTA-(OH)**_**3**_ homopolymers as shown by SLS, SANS, and cryo-TEM. By utilizing the modularity of supramolecular copolymerization, the system was further expanded to comprise the surface-anchoring monomer **BTA-Ba** and yielded multicomponent supramolecular copolymers. The H/D exchange of **BTA-DNP**_**3**_ within the copolymers showed a similar profile as compared to **BTA-(OH)**_**3**_, indicating that **BTA-DNP**_**3**_ monomers are indeed incorporated into the supramolecular copolymers. The H/D exchange also revealed that **BTA-DNP**_**3**_ monomers are likely incorporated within less ordered parts of the supramolecular polymers. Analysis of the copolymerization of **BTA-DNP**_**3**_ monomers using MD simulations corroborated the decrease in stacking order upon incorporation of **BTA-DNP**_**3**_ due to back-folding of the DNP moiety. A functional multicomponent system was demonstrated upon the incubation of supramolecular copolymers bearing both **BTA-Ba** and **BTA-DNP**_**3**_ moieties onto SLBs. This revealed that both functional groups in the supramolecular copolymers remain accessible for binding and the supramolecular copolymers specifically interact with anti-DNP antibodies. The system was further expanded by evaluating the exchange of functional monomers present in free fibers into fibers attached to surfaces. The regained ability of the supramolecular polymers anchored on supported lipid bilayers to antibody binding highlights the dynamic nature of the functional BTA monomers. Here, we also demonstrated that this dynamicity present in supramolecular polymers allows for the reorganization and transformation of nonbinding into antibody-binding anchored supramolecular polymers. The combination of multiple functionalities within BTA-based supramolecular copolymers highlights the modularity of supramolecular copolymerization, which, in combination with their inherent dynamicity, opens new avenues for adaptive antibody-binding materials.

## Supporting Information Available

Additional synthetic procedures, NMR spectra of final compounds, mass spectra of final compounds, SANS data analysis, HDX-MS data analysis, computational details, details on analysis of TIRF images, additional cryo-TEM images, SANS spectra, UV–vis spectra, HDX-MS curves, and additional TIRF images on SLB formation (PDF)
