# Supplementary material for: Antibody-Recruiting Surfaces Using Adaptive Multicomponent Supramolecular Copolymers
Source: Biomacromolecules. 2025 Apr 9;26(5):2971–85. doi: 10.1021/acs.biomac.5c00043 (PMC12076489; doi:10.1021/acs.biomac.5c00043)
Supplement: Supplementary file 1 — bm5c00043_si_001.pdf [file bm5c00043_si_001.pdf]

Supporting Information

Antibody-recruiting surfaces using adaptive multicomponent supramolecular copolymers

Marle E.J. Vleugels[1], Esmee de Korver[1], Simone I.S. Hendrikse[1], Sinan Kardas[2], Shikha Dhiman[1], Bas F.M. de Waal[1], Sandra M.C. Schoenmakers[1], S. Wijker[1], Bruno G. De Geest[3], Mathieu Surin[2], Anja R.A. Palmans[1], and E.W. Meijer[1,4]**

[1]Laboratory of Macromolecular and Organic Chemistry, Institute for Complex Molecular Systems, Eindhoven University of Technology, P.O. Box 513, 5600 MB Eindhoven, The Netherlands

[2]Laboratory for Chemistry of Novel Materials, Center of Innovation and Research in Materials and Polymers, University of Mons–UMONS, Belgium

[3]Department of Pharmaceutics, Ghent University, 9000 Ghent, Belgium

[4]School of Chemistry and RNA Institute, UNSW, Sydney, NSW 2052, Australia

1. Experimental section S1

1.1 Synthetic procedures S1

2. SANS data analysis S7

3. HDX-MS data analysis S8

4. Computational details S10

5. Analysis of TIRF images S14

6. Supporting Figures S15

7. References S25

1. Experimental section

1.1 Synthetic procedures

BTA-DNP$_1$

BTA-NH$_2$ (152 mg, 0.12 mmol, 1 eq) was dissolved in 5 mL dry DCM and 25 µL of TEA was added (0.18 mmol, 1.5 eq), as well as 50 µL methoxyethanol. NHS-EO$_4$-DNP (98 mg, 0.19

mmol, 1.5 eq) was dissolved in 1 mL dry DCM and added dropwise. The solution was stirred for 16 h at RT under Ar. TLC in 9/1 $CHCl_3$/MeOH showed full conversion of BTA-NH_2. 457 mg *Argopore*-NH_2-LL resin was added to capture excess of NHS ester and the solution was stirred for 24h. After addition of 30 mL DCM the resin was removed by filtration and the filtrate transferred to a separation funnel. The organic phase was washed three times with 20 mL 1M NaOH and once with 20 mL brine. The water phase was extracted once with DCM and the combined organic phases were dried over $MgSO_4$. After concentration the crude was co-evaporated with $CHCl_3$ (2 times 20 mL) to remove TEA. The crude product was further purified by dialysis against water (RC membrane, MWCO 1000) followed by reverse phase column chromatography (H_2O/ACN gradient 100/0 to 10/90). The solvent was removed by lyophilization giving a yellow waxy solid. Yield: 39 mg (19%).

^{1}H NMR (400 MHz, $CDCl_3$) δ 9.14 (d, *J* = 2.7 Hz, 1H), 8.87 – 8.75 (m, 1H), 8.38 (s, 3H), 8.27 (dd, *J* = 9.5, 2.7 Hz, 1H), 6.96 (d, *J* = 9.6 Hz, 1H), 6.89 – 6.77 (m, 4H), 3.82 (t, *J* = 5.2 Hz, 2H), 3.77 – 3.50 (m, 62H), 3.50 – 3.37 (m, 14H), 3.18 (s, 2H), 2.45 (t, *J* = 6.1 Hz, 2H), 1.66 – 1.51 (m, 12H), 1.44 – 1.23 (m, 48H). ^{13}C NMR (100 MHz, $CDCl_3$) δ 171.34, 165.84, 148.42, 136.09, 135.24, 130.27, 128.14, 124.30, 114.12, 72.64, 71.56, 70.75, 70.68, 70.59, 70.55, 70.51, 70.42, 70.25, 70.19, 70.01, 69.87, 68.59, 67.28, 61.68, 43.25, 40.38, 39.17, 36.89, 29.56, 29.51, 29.46, 29.43, 29.39, 29.25, 29.23, 26.95, 26.03, 0.00. LC-MS: calculated m/z = 1700 for $C_{86}H_{153}N_7O_{26}$, observed m/z = 568.08 $[M+3H]^{3+}$, 851.50 $[M+2H]^{2+}$, 1700.83 $[M+H]^{+}$

BTA-DNP$_3$

BTA-$(NH_2)_3$ (98 mg, 0.0762 mmol, 1 eq) was dissolved in 5 mL dry DCM and 40 µL of TEA was added (0.259 mmol, 3.4 eq). NHS-EO_4-DNP (137 mg, 0.259 mmol, 3.4 eq) was dissolved in 2 mL dry DCM and added dropwise. The solution was stirred for 16 h at RT under Ar. NMR indicated completion of the reaction and 220 mg *Argopore*-NH_2-LL resin was added to capture excess of NHS ester and the solution was stirred for 24 h. After addition of 30 mL DCM the resin was removed by filtration and the filtrate transferred to a separation funnel. The organic phase was washed three times with 20 mL 1M NaOH, once with 20 mL water and once with 20 mL brine. After concentration the crude was co-evaporated with $CHCl_3$ (2 times 20 mL) to remove TEA. The crude product was further purified by column chromatography (CHCl3/MeOH gradient 100/0 to 80/20). The solvent was removed *in vacuo* giving a yellow waxy solid. Yield: 80 mg (42%).

^{1}H NMR (400 MHz,) δ 9.17 – 9.08 (m, 3H), 8.87 – 8.74 (m, 3H), 8.38 (s, 3H), 8.27 (dd, *J* = 9.5, 2.5 Hz, 3H), 7.00 – 6.79 (m, 9H), 3.82 (t, *J* = 5.2 Hz, 6H), 3.76 – 3.36 (m, 108H), 2.46 (t, *J* = 6.1 Hz, 6H), 1.65 – 1.50 (m, 12H), 1.45 – 1.15 (m, 48H). ^{13}C NMR (100 MHz, $CDCl_3$) δ 171.34, 165.82, 148.42, 136.08, 135.27, 130.51, 130.26, 128.15, 124.30, 114.13, 71.55, 70.75, 70.68, 70.60, 70.58, 70.54, 70.51, 70.41, 70.26, 70.18, 70.02, 69.88, 68.59, 67.29, 43.25, 40.38, 39.17, 36.88, 29.56, 29.52, 29.49, 29.46, 29.42, 29.27, 26.98, 26.04, 0.00. MALDI-TOF-MS: calculated MW = 2525.99 g/mol for $C_{120}H_{201}N_{15}O_{42}$, observed m/z = 2548.47 [M+Na]+.

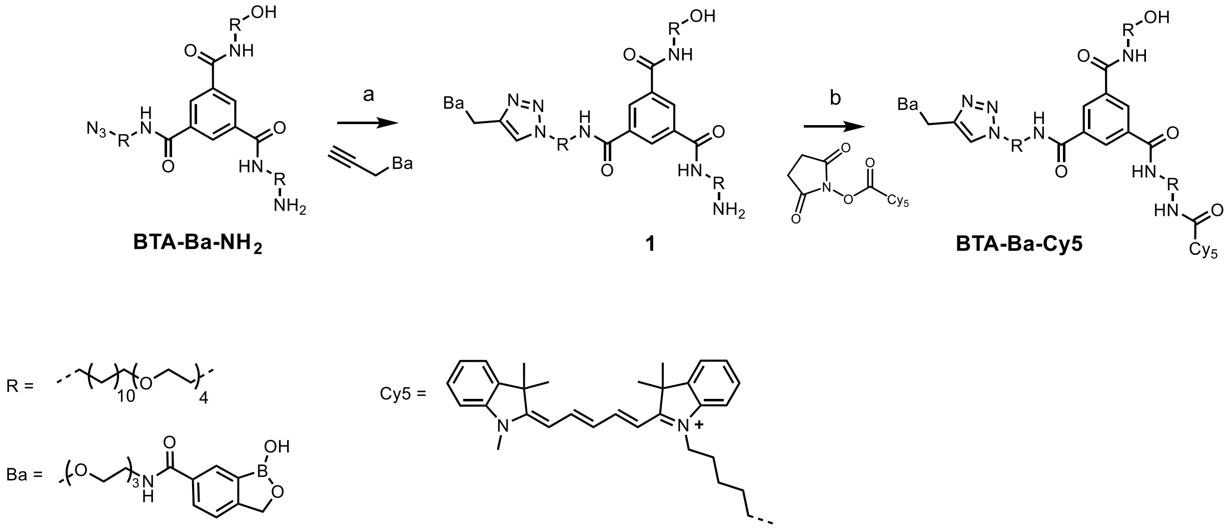

Scheme S1. Synthetic route towards difunctional BTA monomer **BTA-Ba-Cy5**. Reaction conditions: (a) $CuSO_4$, NaAsc, Bim(Py)$_2$, aminoguanidine, DMF/H_2O, quant. (b) DMSO, TEA, 14% (**BTA-Ba-Cy5**).

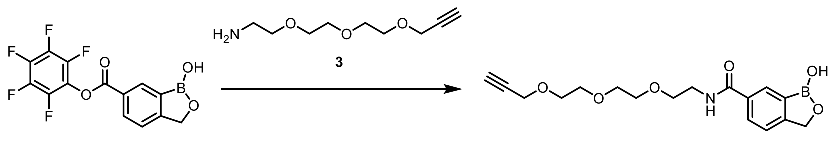

2 4

1-Hydroxy-N-(2-(2-(2-(prop-2-yn-1-yloxy)ethoxy)ethoxy)ethyl)-1,3-dihydrobenzo[c][1,2] oxaborole-6-carboxamide (Ba-alkyne, (4))

Perfluorophenyl 1-hydroxy-1,3-dihydrobenzo[c][1,2]oxaborole-6-carboxylate (PFP-Ba (**2**), 100.4 mg, 291.86 µmol, 1 eq) was stirred with 1 mL DCM under an argon atmosphere. Triethylamine (0.17 mL, 1.21 mmol, 4.14 eq) was added to the turbid mixture which became clear within one minute. A solution of OEG$_3$-alkyne-NH_2 (**3**) (109.3 mg, 583.72 µmol, 2 eq) in 2 mL DCM was added dropwise to the mixture and stirred overnight at room temperature. A TLC analysis (eluents $CHCl_3$/MeOH/acetic acid 90/9/1 v/v, R_f = 0.5, stained with $KMnO_4$) confirmed conversion, and the solvent was removed in vacuo. The crude product was purified

with column chromatography using a Biotage Isolera One column machine (Biotage Sfär 25 g prepacked silica column) by flushing with a solvent mixture of $CHCl_3$/MeOH/acetic acid 90/9/1 v/v. The product was co-evaporated trice with $CHCl_3$. Yield: 100.3 mg (98 %). ^{1}H NMR (400 MHz, DMSO-d_6) δ 9.29 (s, 1H), 8.51 (t, *J* = 5.6 Hz, 1H), 8.22 (s, 1H), 7.93 (dd, *J* = 8.0, 1.7 Hz, 1H), 7.48 (d, *J* = 8.0 Hz, 1H), 5.04 (s, 2H), 4.12 (d, *J* = 2.4 Hz, 2H), 3.54 (d, *J* = 5.3 Hz, 8H), 3.46 – 3.38 (m, 4H), 3.17 (s, 1H). ^{13}C NMR (100 MHz, DMSO) δ 167.07, 157.19, 133.88, 130.06 (d, *J* = 23.7 Hz), 121.68, 80.78, 79.64, 77.56, 70.68 – 68.58 (m), 57.94. ^{19}F NMR (375 MHz, DMSO) δ -162.05 (d, *J* = 22.7 Hz), -165.63 (t, *J* = 22.9 Hz), -172.44. LC-MS: Rt = 3.76 min, calculated m/z = 347.15, observed m/z = 348.17 $[M+H]^+$.

N1-(1-Amino-3,6,9,12-tetraoxatetracosan-24-yl)-N3-(1-(4-(1-(1-hydroxy-1,3 dihydrobenzo[c][1,2]oxaborol-6-yl)-1-oxo-5,8,11-trioxa-2-azadodecan-12-yl)-1H-1,2,3-triazol-1-yl)-3,6,9,12-tetraoxatetracosan-24-yl)-N5-(1-hydroxy-3,6,9,12-tetraoxatetracosan-24-yl)benzene-1,3,5-tricarboxamide (1)

Four reaction cocktails were prepared for the CuAAC click reaction; $CuSO_4$ (50 mM) in MQ, aminoguanidine (200 mM) in MQ, Bim(Py)$_2$ (30 mM) in DMF, and NaAsc (200 mM) in MQ. In this sequence, 200 µL of each cocktail was added to a small sample vial which resulted a coloring sequence; blue, green, and brown, respectively. This brown solution was added to a small sample vial containing N1-(1-amino-3,6,9,12-tetraoxatetracosan-24-yl)-N3-(1-azido-3,6,9,12-tetraoxatetracosan-24-yl)-N5-(1-hydroxy-3,6,9,12-tetraoxatetracosan-24-yl)benzene-1,3,5-tricarboxamide (**BTA-NH$_2$-N$_3$**) (40 mg, 30.47 µmol, 1 eq) and alkyne-Ba (**8**) (12.7 mg, 36.56 µmol, 1.2 eq) in 400 µL DMF. The vial was stirred at room temperature overnight, and LC-MS analysis confirmed reaction conversion. The brown solution was passed through a glass filter filled with 3 cm of silica and flushed with $CHCl_3$/MeOH/IPA 68/30/2 v/v. The product fraction was verified with LC-MS, concentrated in vacuo, dissolved again in water, and freeze-dried to obtain the product as brownish sticky solid (60 mg, ~100%)

LC-MS: Rt = 5.89 min, calculated m/z = 1659.10, observed m/z = 1660.17 $[M+H]^+$, 830.75 $[M+2H]^{2+}$, 554.33 $[M+3H]^{3+}$.

BTA-Ba-Cy5

N1-(1-Amino-3,6,9,12-tetraoxatetracosan-24-yl)-N3-(1-(4-(1-(1-hydroxy-1,3 dihydrobenzo[c][1,2]oxaborol-6-yl)-1-oxo-5,8,11-trioxa-2-azadodecan-12-yl)-1H-1,2,3-triazol-1-yl)-3,6,9,12-tetraoxatetracosan-24-yl)-N5-(1-hydroxy-3,6,9,12-tetraoxatetracosan-24-yl)benzene-1,3,5-tricarboxamide (**1**) (13.68 mg, 8.24 µmol, 1.1 eq), NHS-coupled cyanine-

5 dye (5 mg, 6.20 µmol, 1 eq), TEA (4.2 µL, 30 µmol, 4 eq) were dissolved in 300 µL DMSO in a sample vial. The reaction was stirred overnight at room temperature shielded from light. The crude was loaded on a Biotage Isolera One column machine (Biotage Sfär 6 g prepacked silica column) with a gradient of $CHCl_3$/MeOH/isopropylamine 100/0/0 to 87.5/10/2.5 v/v/v. Further purification was done on a C18 reverse phase column using a Biotage Isolera One column machine (Biotage Sfär 6 g prepacked silica C18 column) with a gradient of water/ACN 60/40 to 100/0 v/v. The product fraction was verified with LC-MS, and freeze-dried to obtain the product as a blue sticky solid (1.8 mg, 14%).

LC-MS: Rt = 5.05 min, calculated m/z 2125.40, calculated m/z internal ester 2108.40, observed m/z = 1069.42 $[\text{internal ester + MeOH + H}]^{2+}$, 705.92 $[\text{internal ester + 2H}]^{3+}$, 529.75 $[\text{internal ester + 3H}]^{4+}$

Additional note on the internal ester formation for benzoxaborole functionalized BTAs

During the synthesis of BTA derivatives presenting benzoxaborole, mass analysis (MALDI-TOF-MS as well as LC-MS) indicates the presence of boronic ester derivatives, wherein the peripheral alcohol on the Ba and the BTA side chains undergo an intramolecular reaction (Figure S1A). The presence of the intramolecular BTA boronic ester derivate is undesirable, as the boronic acid would not be available for binding with sialic acid. Therefore, an ^{1}H NMR (in DMSO-d_6) was measured to rule out this boronic ester formation. Figure S1B shows the spectrum of the BTA-Ba product wherein the signals of both alcohols integrate correctly. A small-scale proton-deuterium exchange (HDX) experiment was performed by adding a drop of D_2O to the sample in DMSO-d_6. The most labile protons were exchanged for a deuterium, meaning that these proton peaks disappeared in the spectrum (Figure S1C). Hence, these protons were present in the sample and the product was formed. The formation of the boronic ester derivate occurs during ionization, or due to the slightly acidic environment during LC-MS due to the presence of formic acid in the mobile phase.

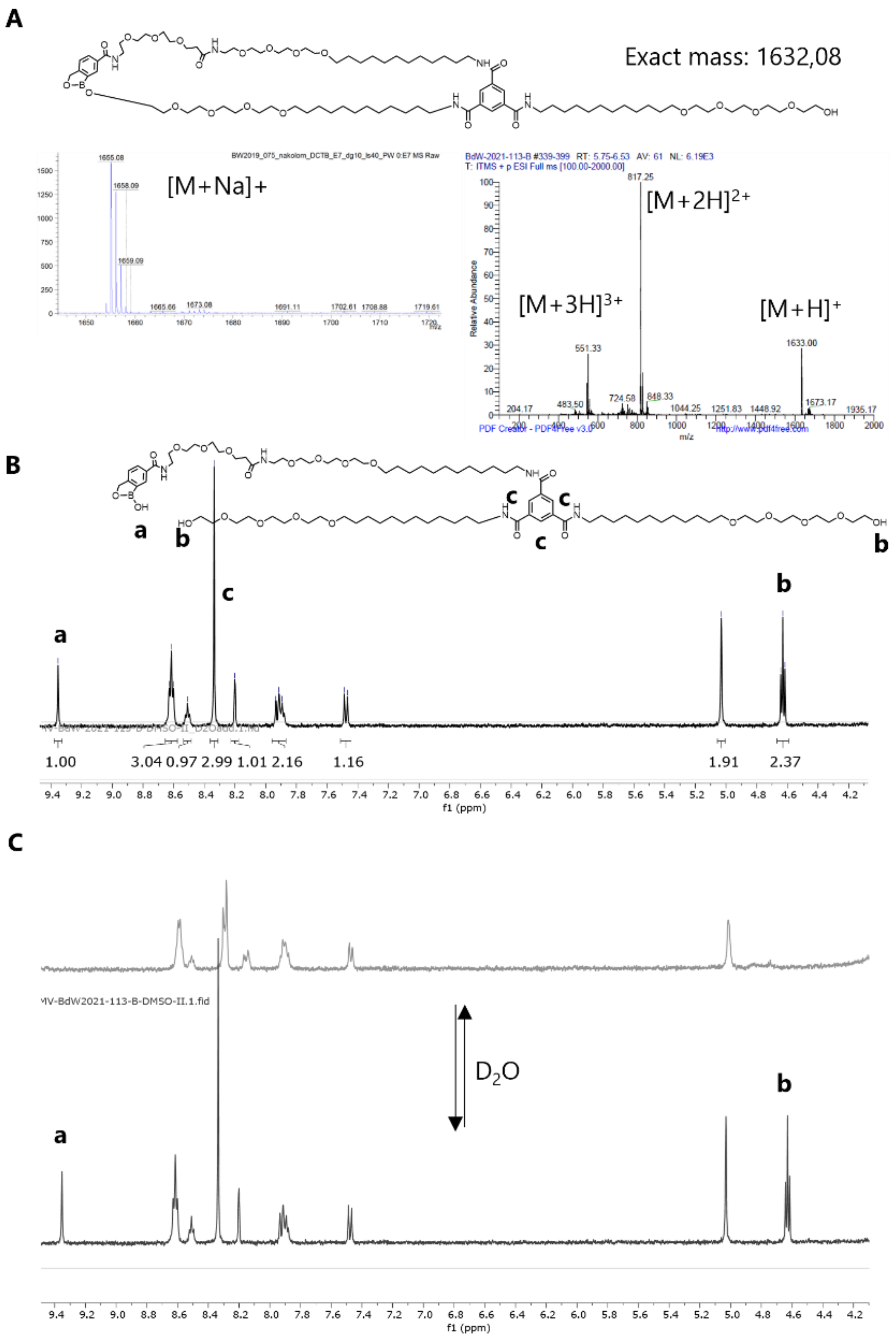

Figure S1 (A) Boronic ester derivative formation during analysis of **BTA-Ba**, with corresponding mass spectra from MALDI-TOF-MS and LC-MS. (B) ^{1}H NMR spectrum of **BTA-Ba** in DMSO-d_6. (C) ^{1}H NMR spectra of **BTA-Ba** in DMSO-d_6 (black, bottom) with a drop of D_2O (grey, top) wherein the peripheral hydrogens exchange for deuterium and the signals of a and b disappear.

2. SANS data analysis

The overall scattering curve intensities, I(q), are defined by the product of the form factor, P(q), and the structure factor S(q) plus incoherent scattering contributions (background):

$$I(q) = N(\Delta\rho)^2 * P(q) * S(q) + background$$

Where N is the number density of scattering objects, $\Delta\rho$ is the contrast term of the scattering length density difference between the objects and the solvent and q the scattering vector.

At low concentrations S(q) ~1, thus the scattering profile is mainly determined by $P(q)$. Form factor fits were performed using SasView ® 5.0.5 software. The scattering length density of D_2O was fixed to 6.35 $*10^{-6}$ Å^{-2}, the scattering length densities for **BTA-(OH)$_3$** monomers were calculated in the software and fixed to 5.39$*10^{-6}$ Å^{-2}

The curves were fit with the built-in elliptical cylinder model (equation 1)

$$I(q) = \frac{1}{Vcyl}\int d\psi \int d\phi \int p(\theta,\phi,\psi)F^2(q,\alpha,\psi)\sin(\alpha)\,d\alpha \quad (1)$$

With the functions

$$F(q,\alpha,\psi)\sin(\alpha) = 2\frac{J_1(a)\sin(b)}{ab}$$

where
$$a = qr'\sin(\alpha)$$

$$b = q\frac{L}{2}\cos(\alpha)$$

$$r' = \frac{r_{minor}}{\sqrt{2}}\sqrt{(1+v^2)+(1-v^2)\cos(\psi)}$$

and the angle ψ is defined as the orientation of the major axis of the ellipse with respect to the vector q. The angle α is the angle between the axis of the cylinder and q.

For 1D scattering, with no preferred orientation, the form factor is averaged over all possible orientations and normalized by the particle volume

$$P(q) = scale < F^2 > /V$$

Table S1. Parameters obtained from SANS fits as shown in Figure 3. [a] Short cross-section diameter of the cylinder. [b] Length of the cylinders.

Assembly	Fit	2*a (Å)[a]	Axis ratio	L (Å)[b]	X^2
**BTA-(OH)$_3$**	Elliptical cylinder	45.6	1.87	>>2000	7.5
**BTA-DNP$_3$ 2.5%**	Elliptical cylinder	45.6	1.85	>>2000	8.3

3. HDX-MS data analysis

For the copolymer the H/D exchange was followed over time and the percentage of the different deuterated isotopes was calculated. The distribution with two sodium ions was used for the calculations of **BTA-(OH)$_3$** and the distribution with 3 sodium ions was used for **BTA-DNP$_3$**. Isotope distributions were calculated with IsoPro Software. The data for the homopolymer of **BTA-(OH)$_3$** originates from previous work of our group.[6]

The following set of equations was used for the isotope correction of **BTA-(OH)$_3$** in the copolymer:

$I_{BTA1D\_c} = I_{667.46}$

$I_{BTA2D\_c} = I_{667.96} - 0.80 I_{BTA1D\_c}$

$I_{BTA3D\_c} = I_{668.47} - 0.80 I_{BTA2D\_c} - 0.36 I_{BTA1D\_c}$

$I_{BTA4D\_c} = I_{668.97} - 0.80 I_{BTA3D\_c} - 0.36 I_{BTA2D\_c} - 0.11 I_{BTA1D\_c}$

$I_{BTA5D\_c} = I_{669.47} - 0.80 I_{BTA4D\_c} - 0.36 I_{BTA3D\_c} - 0.11 I_{BTA2D\_c} - 0.03 I_{BTA1D\_c}$

$I_{BTA6D\_c} = I_{669.97} - 0.80 I_{BTA5D\_c} - 0.36 I_{BTA4D\_c} - 0.11 I_{BTA3D\_c} - 0.03 I_{BTA2D\_c} - 0.01 I_{BTA1D\_c}$

With $I_{667.46}$, $I_{667.96}$, $I_{668.47}$, $I_{668.97}$, $I_{669.47}$ and $I_{669.97}$ the intensity at m/z = 667.46, 667.96, 668.47, 668.97, 669.47 and 669.97, respectively.

The following set of equations was used to correct for 1 vol% H_2O in the calculations for **BTA-(OH)$_3$** in the copolymer:

$I_{BTA1D} = I_{BTA1D\_c} - 1.0\times10^{-9} I_{BTA6D\_c} - 3.1\times10^{-4} I_{BTA3D\_c}$

$I_{BTA2D} = I_{BTA2D\_c} - 1.58\times10^{-7} I_{BTA6D\_c} - 3.04\times10^{-2} I_{BTA3D\_c}$

$I_{BTA3D} = I_{BTA3D\_c} - 2.08\times10^{-5} I_{BTA6D\_c} + \left(3.1\times10^{-4} + 3.04\times10^{-2}\right) I_{BTA3D\_c}$

$I_{BTA4D} = I_{BTA4D\_c} - 1.54\times10^{-3} I_{BTA6D\_c}$

$I_{BTA5D} = I_{BTA5D\_c} - 6.08\times10^{-2} I_{BTA6D\_c}$

$I_{BTA6D} = I_{BTA6D\_c} + \left(1.0\times10^{-9} + 1.58\times10^{-7} + 2.08\times10^{-5} + 1.54\times10^{-3} + 6.08\times10^{-2}\right) I_{BTA6D\_c}$

The percentage of the deuterated analogues for **BTA-(OH)$_3$** can be calculated with:

$$\%BTAnD = \frac{I_{BTAnD}}{\sum_{k=1}^{6} I_{BTAkD}} \times 100\%$$

The following set of equations was used for the isotope correction of **BTA-DNP$_3$** in the copolymer:

$I_{BTA1D\_c} = I_{864.81}$

$I_{BTA2D\_c} = I_{865.15} - 1.43 I_{BTA1D\_c}$

$I_{BTA3D\_c} = I_{865.48} - 1.43 I_{BTA2D\_c} - 1.11 I_{BTA1D\_c}$

$I_{BTA4D\_c} = I_{865.82} - 1.43I_{BTA3D\_c} - 1.11I_{BTA2D\_c} - 0.60I_{BTA1D\_c}$

$I_{BTA5D\_c} = I_{866.15} - 1.43I_{BTA4D\_c} - 1.11I_{BTA3D\_c} - 0.60I_{BTA2D\_c} - 0.26I_{BTA1D\_c}$

$I_{BTA6D\_c} = I_{866.49} - 1.43I_{BTA5D\_c} - 1.11I_{BTA4D\_c} - 0.60I_{BTA3D\_c} - 0.26I_{BTA2D\_c} - 0.09I_{BTA1D\_c}$

$I_{BTA7D\_c} = I_{866.82} - 1.43I_{BTA6D\_c} - 1.11I_{BTA5D\_c} - 0.60I_{BTA4D\_c} - 0.26I_{BTA3D\_c} - 0.09I_{BTA2D\_c} - 0.031I_{BTA1D\_c}$

$I_{BTA8D\_c} = I_{867.15} - 1.43I_{BTA7D\_c} - 1.11I_{BTA6D\_c} - 0.60I_{BTA5D\_c} - 0.26I_{BTA4D\_c} - 0.09I_{BTA3D\_c} - 0.031I_{BTA2D\_c} - 0.008I_{BTA1D\_c}$

$I_{BTA9D\_c} = I_{867.49} - 1.43I_{BTA8D\_c} - 1.11I_{BTA7D\_c} - 0.60I_{BTA6D\_c} - 0.26I_{BTA5D\_c} - 0.09I_{BTA4D\_c} - 0.031I_{BTA3D\_c} - 0.008I_{BTA1D\_c} - 0.002I_{BTA2D\_c}$

With $I_{864.81}$, $I_{865.15}$, $I_{865.48}$, $I_{865.82}$, $I_{866.15}$, $I_{866.49}$, $I_{866.82}$, $I_{867.15}$ and $I_{867.49}$ the intensity at m/z = 864.81, 865.15, 865.82, 866.15, 866.49, 866.82, 867.15 and 867.49, respectively.

The following set of equations was used to correct for 1 vol% H_2O in the calculations for **BTA-DNP$_3$** in the copolymer:

$I_{BTA1D} = I_{BTA1D\_c} - 1.0\times10^{-15}I_{BTA9D\_c} - 6.42\times10^{-10}I_{BTA6D\_c}$

$I_{BTA2D} = I_{BTA2D\_c} - 3.96\times10^{-13}I_{BTA9D\_c} - 1.58\times10^{-7}I_{BTA6D\_c}$

$I_{BTA3D} = I_{BTA3D\_c} - 9.11\times10^{-11}I_{BTA9D\_c} - 2.08\times10^{-5}I_{BTA6D\_c}$

$I_{BTA4D} = I_{BTA4D\_c} - 1.35\times10^{-8}I_{BTA9D\_c} - 1.54\times10^{-3}I_{BTA6D\_c}$

$I_{BTA5D} = I_{BTA5D\_c} - 1.33\times10^{-6}I_{BTA9D\_c} - 6.08\times10^{-2}I_{BTA6D\_c}$

$I_{BTA6D} = I_{BTA6D\_c} - 8.75\times10^{-5}I_{BTA9D\_c} + \left(6.42\times10^{-10}+1.58\times10^{-7}+2.08\times10^{-5}+1.54\times10^{-3}+6.08\times10^{-2}\right)I_{BTA6D\_c}$

$I_{BTA7D} = I_{BTA7D\_c} - 3.70\times10^{-3}I_{BTA9D\_c}$

$I_{BTA8D} = I_{BTA8D\_c} - 9.12\times10^{-2}I_{BTA9D\_c}$

$$I_{BTA9D} = I_{BTA9D\_c} + \begin{pmatrix} 1.0\times10^{-15}+3.96\times10^{-13}+9.11\times10^{-11}+1.35\times10^{-8}+1.33\times10^{-6}+8.75\times10^{-5} \\ +3.70\times10^{-3}+9.12\times10^{-2}3.1\times10^{-4}+3.04\times10^{-2} \end{pmatrix} I_{BTA9D\_c}$$

The percentage of the deuterated analogues for **BTA-DNP$_3$** can be calculated with:

$$\%BTAnD = \frac{I_{BTAnD}}{\sum_{k=1}^{9} I_{BTAkD}} \times 100\%$$

Fitting of HDX-MS curves

The data for the fully deuterated **BTA-(OH)$_3$** and **BTA-DNP$_3$** was plotted as a function of time (Figure 7B, Figure S2) and the data was plotted with a bi-exponential growth function with Origin 2015. The pre-defined function 'ExpGro2' was used for the fit:

$$y = y0 + A_{fast}\exp\left(\frac{x}{t_{fast}}\right) + A_{slow}\exp\left(\frac{x}{t_{slow}}\right)$$

with y the dependent variable (percentage of deuterated molecules), x the independent variable (time) and y0, A_{fast}, A_{slow}, t_{fast}, and t_{slow} the variables that were varied during the fitting. The offset, y0, can be related to the maximum percentage of deuterated molecules that can be reached, A_{fast} and A_{slow} describe the contribution of the fast and slow component and t_{fast} and t_{slow} describe the time constants of the fast and slow component. The rate constants, k, were calculated from the time constants with:

$k_{fast} = 1/t_{fast}$ and $k_{slow} = 1/t_{slow}$

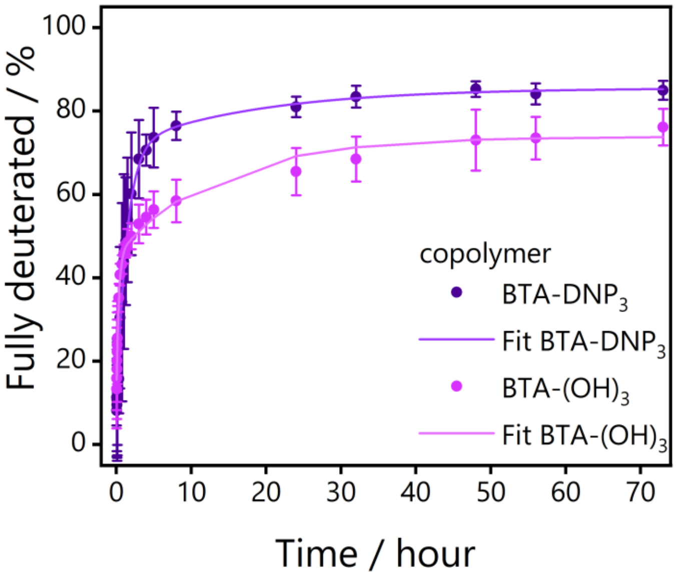

Figure S2. HDX-MS curves of after 100 times dilution of an aqueous sample into D_2O (initial c = 500 µM, after dilution c = 5 µM, T = room temperature) of **BTA-(OH)$_3$** and **BTA-DNP$_3$** monomers within a **BTA-DNP$_3$** : **BTA-Ba** : **BTA-(OH)$_3$** (2.5 : 1 : 96.5) copolymer. The graphs show the amount of fully deuterated monomers as a function of time. The error bars represent the standard deviation calculated from three separate experiments. The lines represent a bi-exponential growth function that was used to fit the data.

4. Computational details

All-atom MD simulations were performed using the GPU version of the AMBER16 package.[7] The molecular models for the BTA monomers were built and parametrized with the 'general AMBER force field (GAFF)'.[8] The partial atomic charges of the monomers were calculated using the semiempirical AM1-BCC model with the *antechamber* module.[9,10] The atomistic models for the supramolecular fibers were built from the 18 pre-stacked cores previously optimized in water with OPLS3 force field into Maestro software (Schrödinger release 2022-3). This model of 18 pre-stacked cores was built such that the hydrogen-bonds are pre-arranged in a "zig-zag" fashion. Then, three extended lateral chains, C_{12}-EG_4-OH_3 for **BTA-OH$_3$** monomers and C_{12}-EG_4-DNP_3 for **BTA-DNP$_3$** monomers, were added around the same starting

core geometry to each BTA core within the Avogadro software.[11] Following this approach, five fibers containing 0, 1 and 6 **BTA-DNP$_3$** monomers with different monomer sequences were built (nBTA, nBTA-5DNP$_3$, nBTA-14DNP$_3$, nBTA-*ran*-DNP$_3$, nBTA-*block* -DNP$_3$, see Figure S2). Molecular mechanics calculations were performed in two steps to optimize the geometry of the fibers. For this, 10,000 steps of energy minimization were performed in the gas phase (i.e., no solvent) to avoid unphysically large forces, consisting in 1,000 steps of the steepest descent algorithm, followed by 9,000 cycles of the conjugate gradient. Then, this phase was repeated in implicit water using the General Born solvation model[12] (ε = 78.5) to remove any steric clashes. After this initial energy minimization phase, the BTA fibers underwent 500 ns of MD simulations. In order to conduct relevant conformational sampling on the sub-microsecond timescale with reasonable computational resources, the MD simulations were achieved in implicit water using the General Born solvation model (ε e = 78.5). Particle velocities in each direction were randomly assigned using the Maxwell-Boltzmann velocity distribution function. The temperature in all simulations was set to the experimental temperature of 20 °C (293 K) and controlled by the Langevin thermostat with a coupling constant of 1.0 ps and combined with a pseudo-random seed generator.[13] Non-bonded interactions were calculated with a virtual infinite cut-off. All MD simulations used a timestep of 2.0 fs and frames were collected at 1.0 ns interval for a total simulation length of 500 ns, resulting in a set of 500 conformations. The resulted trajectories were visualized using the VMD software package[14] and rendering of MD snapshots was performed using PyMOL.[15]

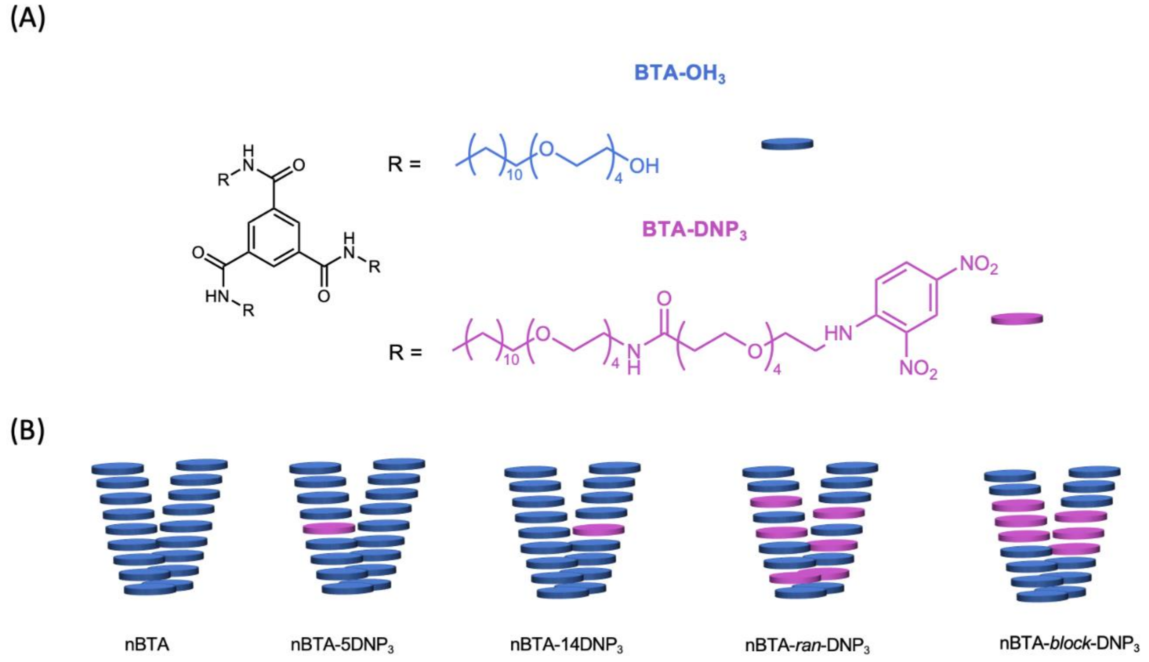

Figure S3 (A) Chemical structures of the two BTA monomers investigated in this study. (B) Schematic representation of the 18-units long fibers containing 0, 1 and 6 **BTA-DNP$_3$** monomers with different monomer sequences.

Analyses of the MD trajectories

The analyses of the trajectories were performed using the *cpptraj* module available in AmberTools.[16] The average length of the helices in the fibers corresponds to the average distance between the center-of-mass of the first and last BTA cores for each helix. The radial distribution function (g(r)) of the distances between the BTA cores was calculated using a bin spacing of 0.1 Å and default density value for normalization (default 0.033456 molecules·Å^{-3}). This data was averaged over all monomers in the fibers. Intermolecular hydrogen-bonds between BTAs were identified using a donor (D)-acceptor (A) distance cutoff of 3.5 Å and no A-H-D angle cutoff to prevent missing any possible hydrogen-bonds. The degree of extension of the side-chains corresponds to the average distance between the center-of-mass of the BTA core and the terminal oxygen atom of tetra(ethylene glycol) tail for **BTA OH$_3$** monomers and between the center-of-mass of the BTA core and the nitrogen atom of the amide of the side-chains or the nitrogen atom of the amine of the side-chains for **BTA-DNP$_3$** monomers (Figure S4-6). The reported distances were averaged over the entire simulation time and over the three side-chains.

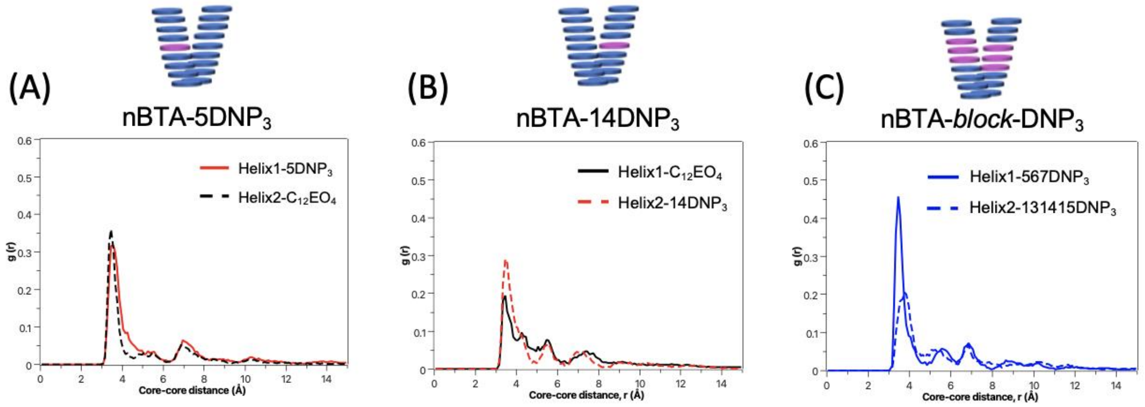

Figure S4. Radial distribution functions g(r) of the neighbor BTA cores along the two helices in the (A) nBTA-5DNP$_3$, (B) nBTA-14DNP$_3$, and (C) nBTA-block-DNP$_3$ copolymers.

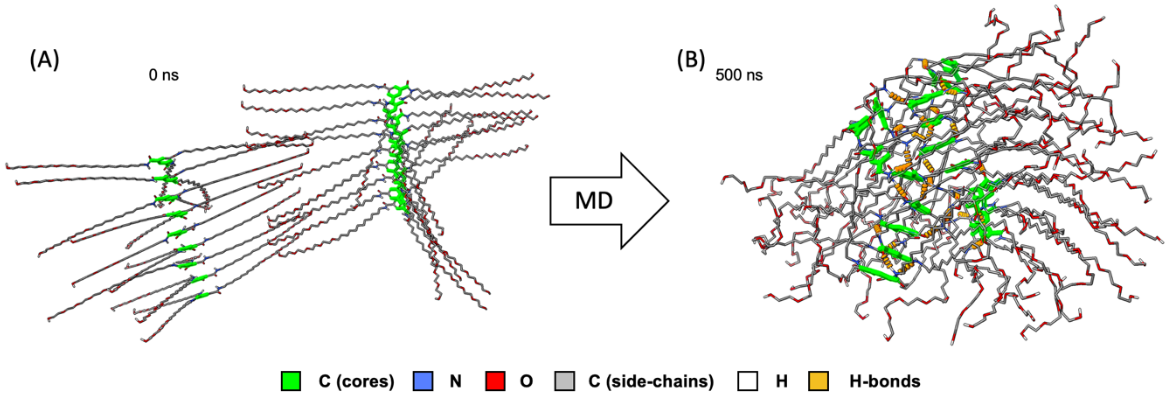

Figure S5 (A) Starting from a geometry of optimized BTA cores with extended side-chains, the geometry of the supramolecular fiber formed by **BTA-OH$_3$** monomers was optimized by energy-minimization to be used as starting structure for MD simulations on a 500 ns timescale. (B) Last snapshot of MD simulations (at 500ns) of the pure **BTA-OH$_3$** homo-assembly, in sticks representation. The carbon atoms of the BTA cores are depicted in green, the nitrogen and the oxygen of the central amides are represented in blue and red, respectively, the carbon atoms of the side-chains are shown in gray and the polar hydrogen atoms in white. The hydrogen bonds between the central amides are highlighted in yellow.

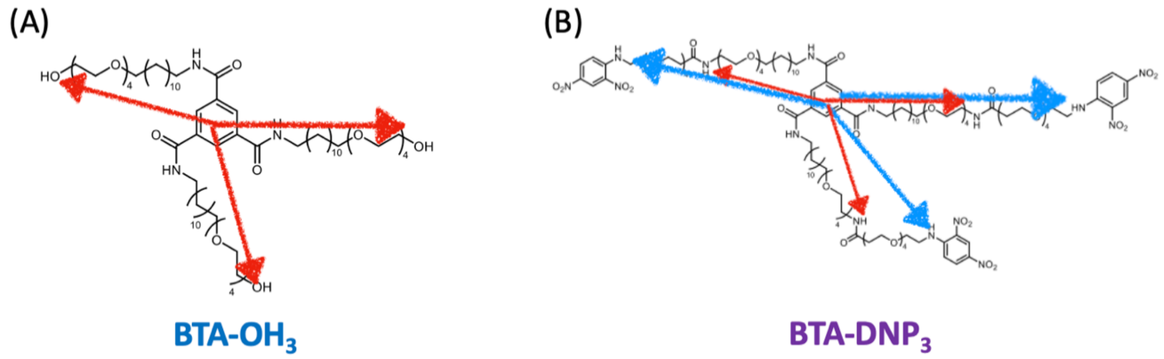

Figure S6 Scheme showing the selected atoms to compute the degree of extension of the side-chains as the average distances (A) between the center-of-mass of the BTA core and the oxygen atom of the tetra(ethylene glycol) tail for **BTA-OH$_3$** monomers and (B) between the center-of-mass of the BTA core and the nitrogen atom of the amide of the side-chains or the nitrogen atom of the amine of the side-chains for **BTA-DNP$_3$** monomers.

5. Analysis of TIRF images

To analyze the relative amount of antibody bound to BTA fibers, 2 channel TIRF images were acquired (488 and 647) and imported into ImageJ. The 647 channel was used to create a mask by first binarizing the image using the automatic thresholding in ImageJ. The binarized image is divided over itself to create a mask of the 647 channel in which the background is “not a number (NaN)”. The 488 channel was multiplied with this mask to only take the antibody bound to the BTA fibers into account and the total intensity of the image was measured. This was done for at least 10 images and the intensities were averaged. To compare **BTA-DNP$_1$** and **BTA-DNP$_3$** bearing assemblies, the intensity of the background was subtracted by inverting the mask of the 647 channel to obtain a mask of the background surrounding the BTA fibers, as this was not constant for all images. The 488 channel was multiplied with this inverted mask and the total intensity of the image was measured. The total intensity of the background was subtracted from the BTA fiber intensity to obtain relative amounts of antibody bound.

To analyze the amount of BTA fibers adsorbed to the surface (Figure S21), the same procedure as above was applied but the mask was multiplied with the 647 channel instead of the 488 channel.

6. Supporting Figures

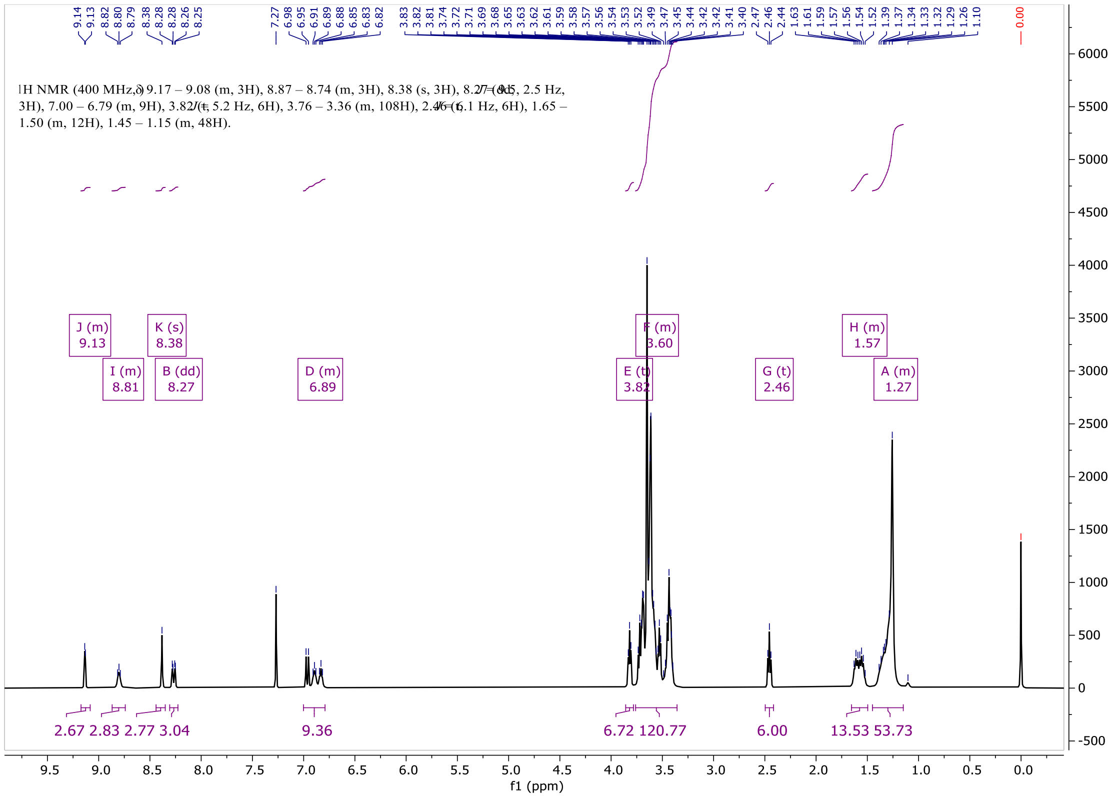

Figure S7 ^{1}H NMR spectrum of **BTA-DNP3** in $CDCl_3$.

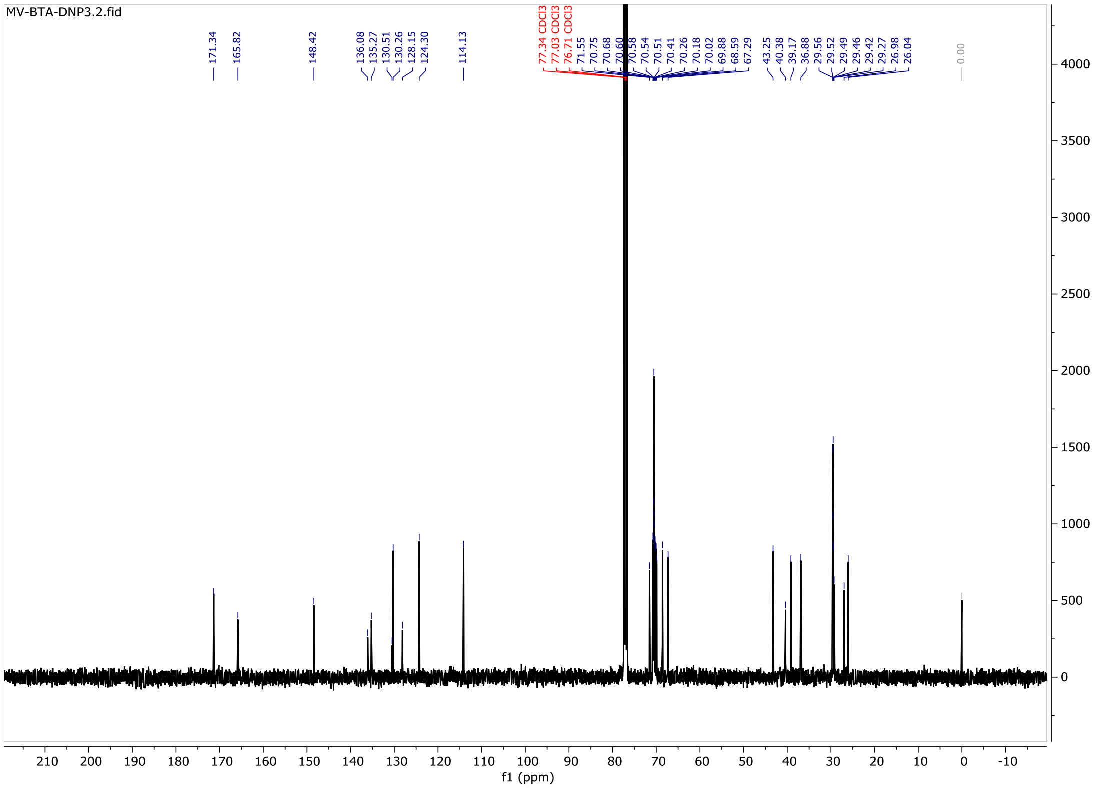

Figure S8 ^{13}C NMR spectrum of **BTA-DNP3** in $CDCl_3$.

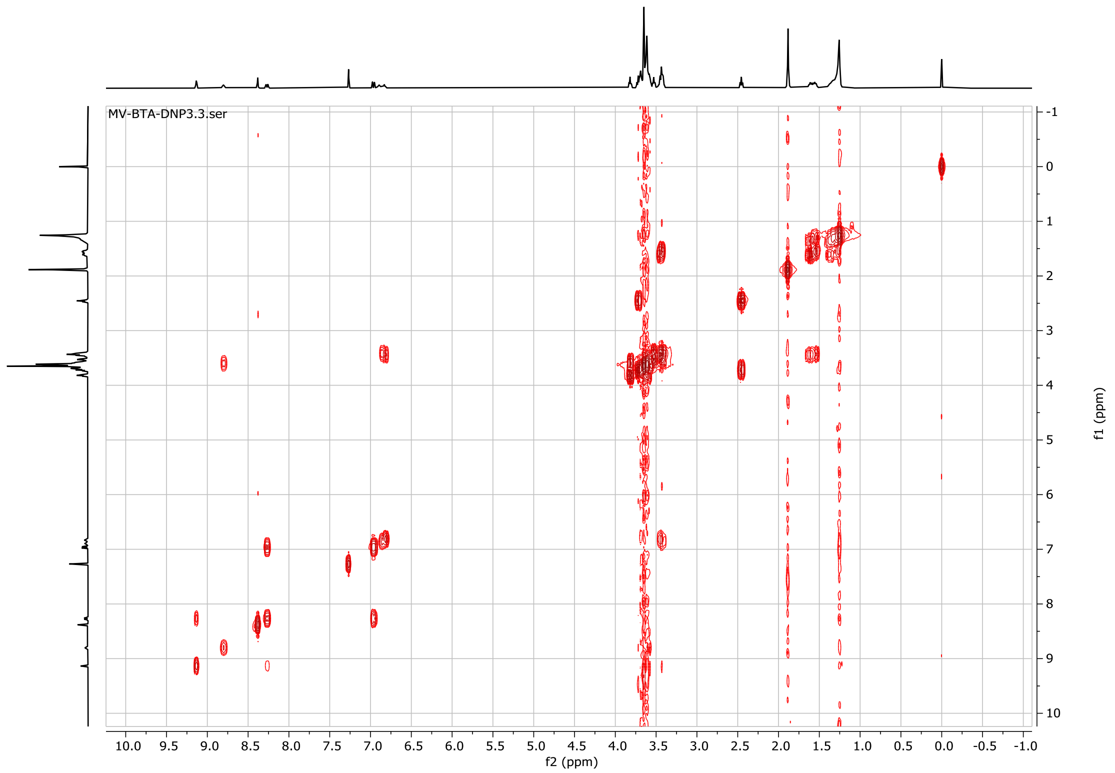

Figure S9 COSY spectrum of **BTA-DNP$_3$** in $CDCl_3$.

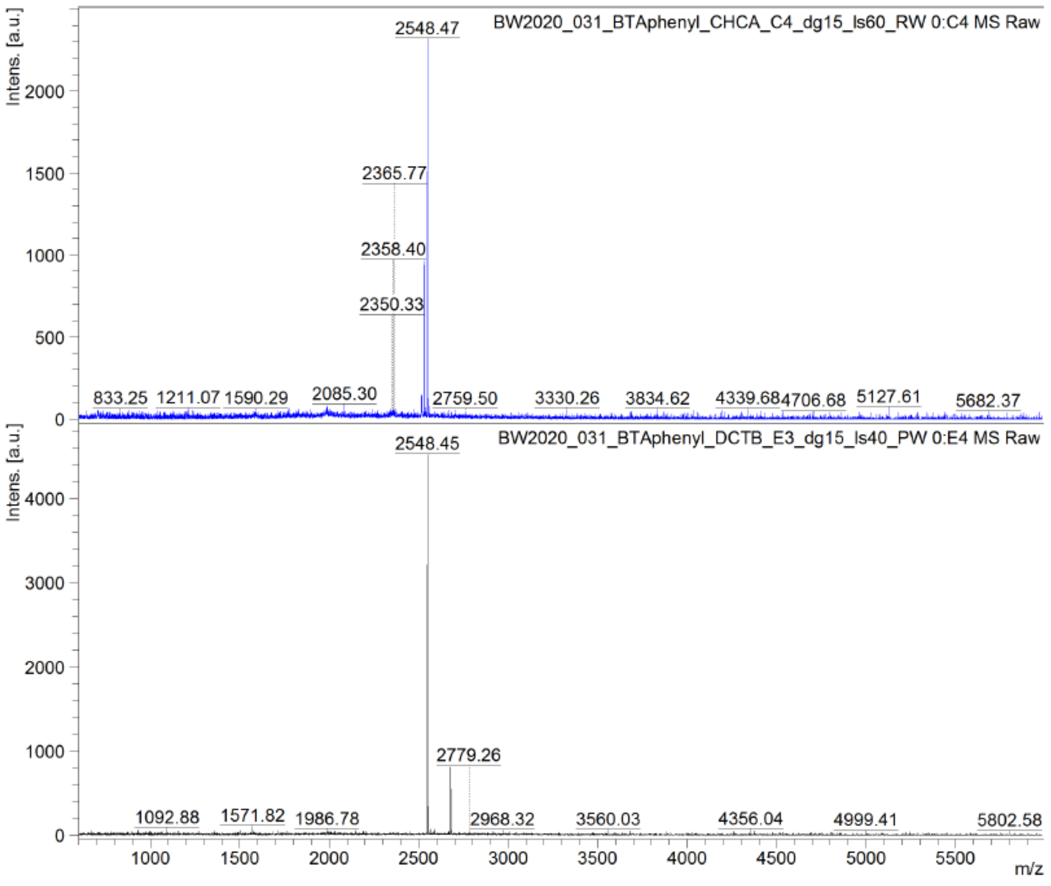

Figure S10 MALDI-TOF-MS spectrum of **BTA-DNP$_3$**.

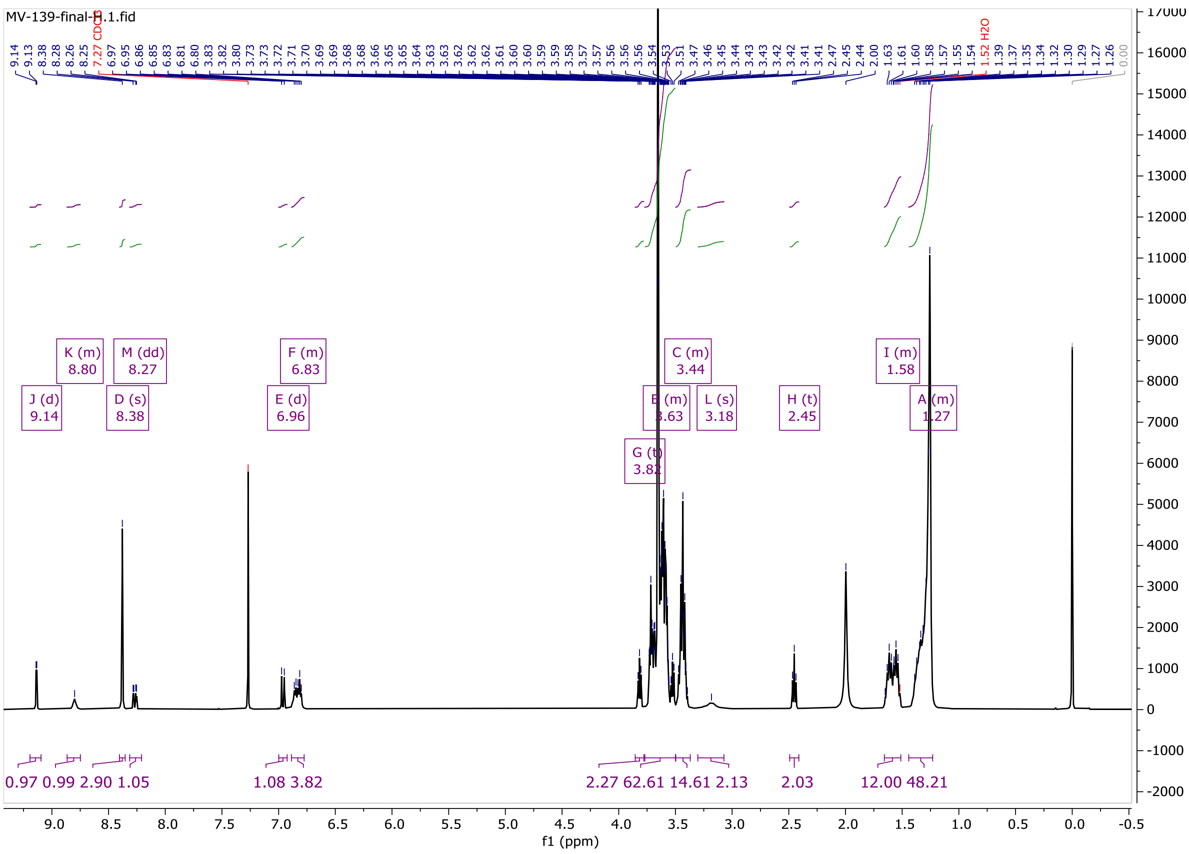

Figure S2 ^{1}H NMR spectrum of **BTA-DNP$_1$** in $CDCl_3$.

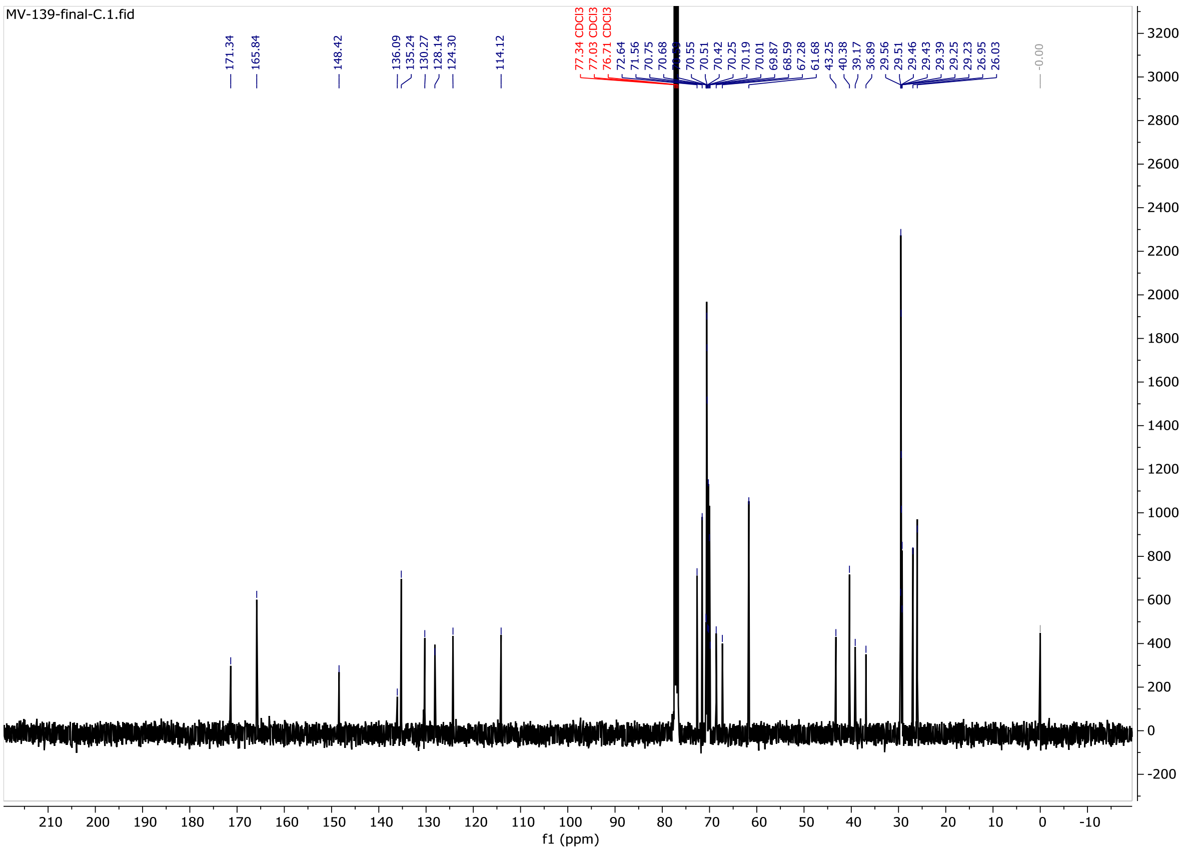

Figure S3 ^{13}C NMR spectrum of **BTA-DNP$_1$** in $CDCl_3$.

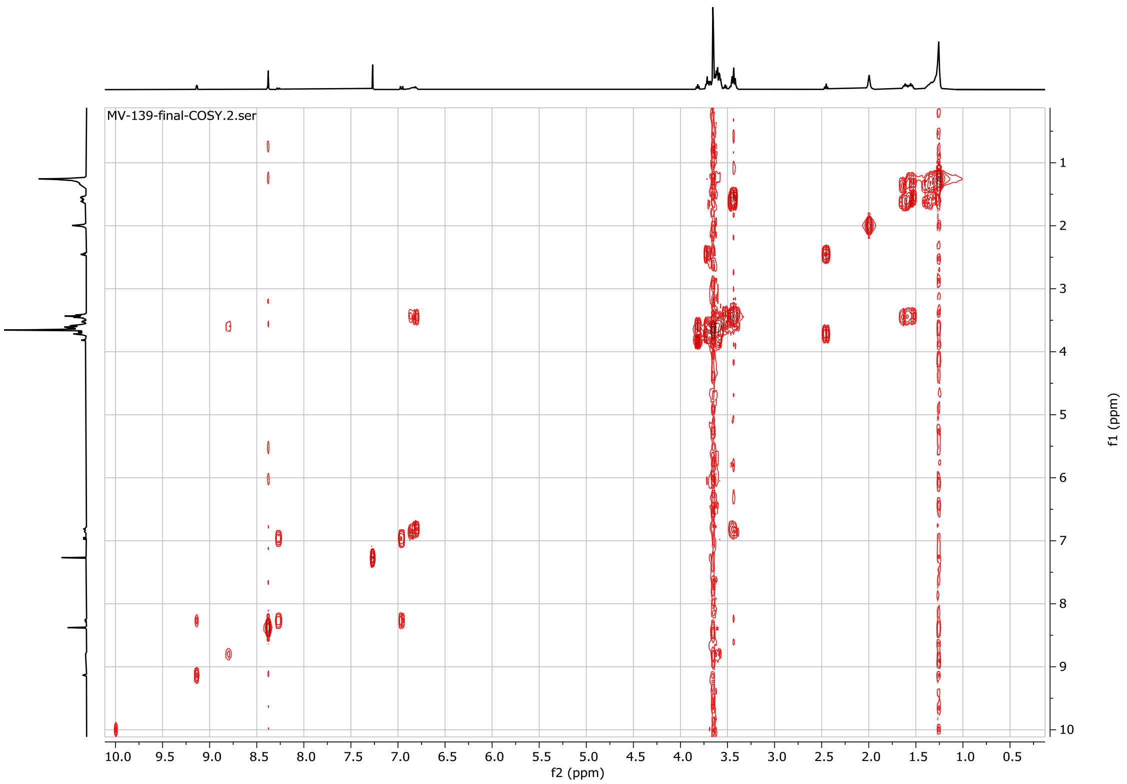

Figure S4 COSY of **BTA-DNP$_1$** in $CDCl_3$.

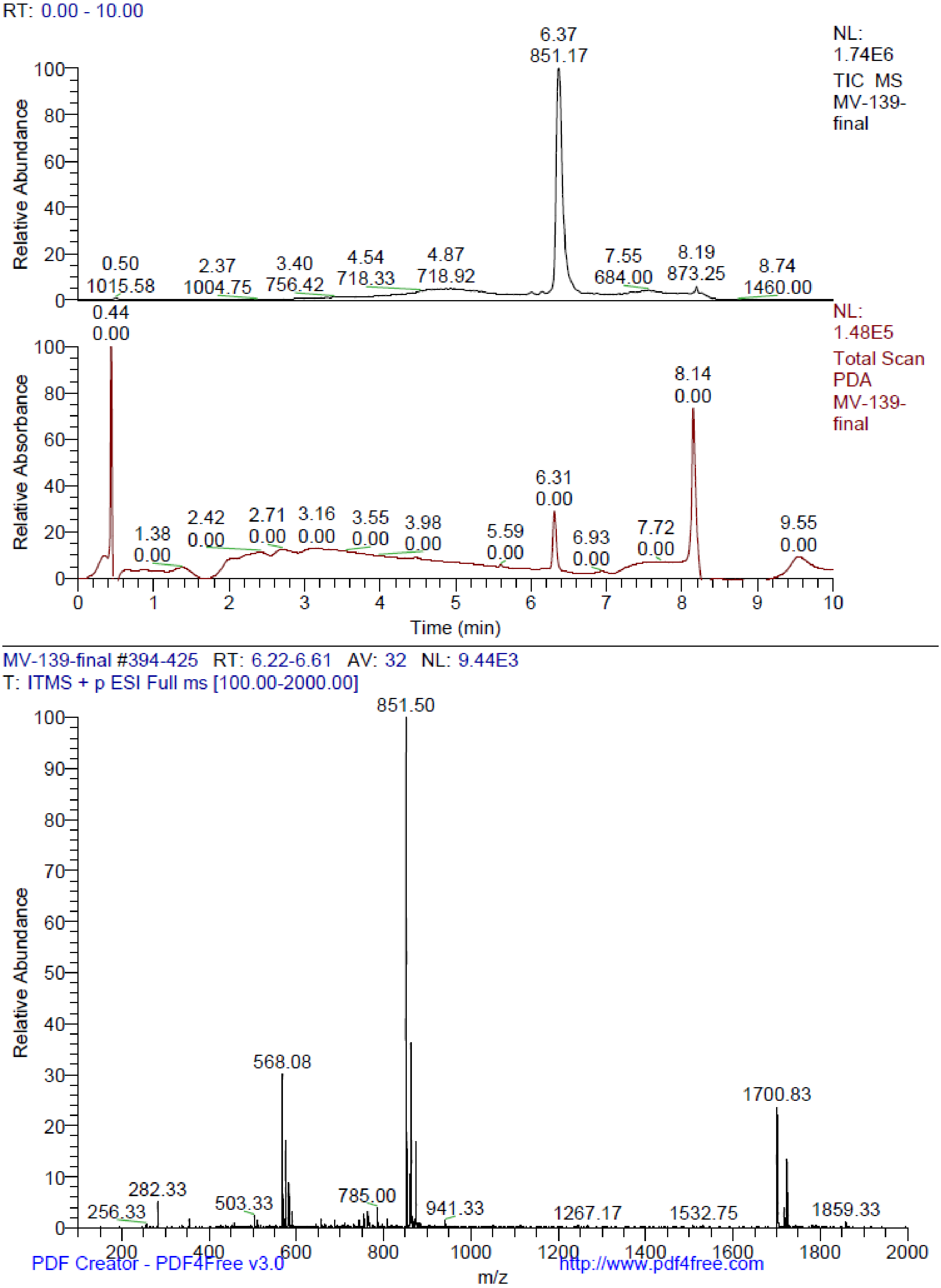

PDF Creator - PDF4Free v3.0
http://www.pdf4free.com

Figure S5 LC-MS of **BTA-DNP$_1$**.

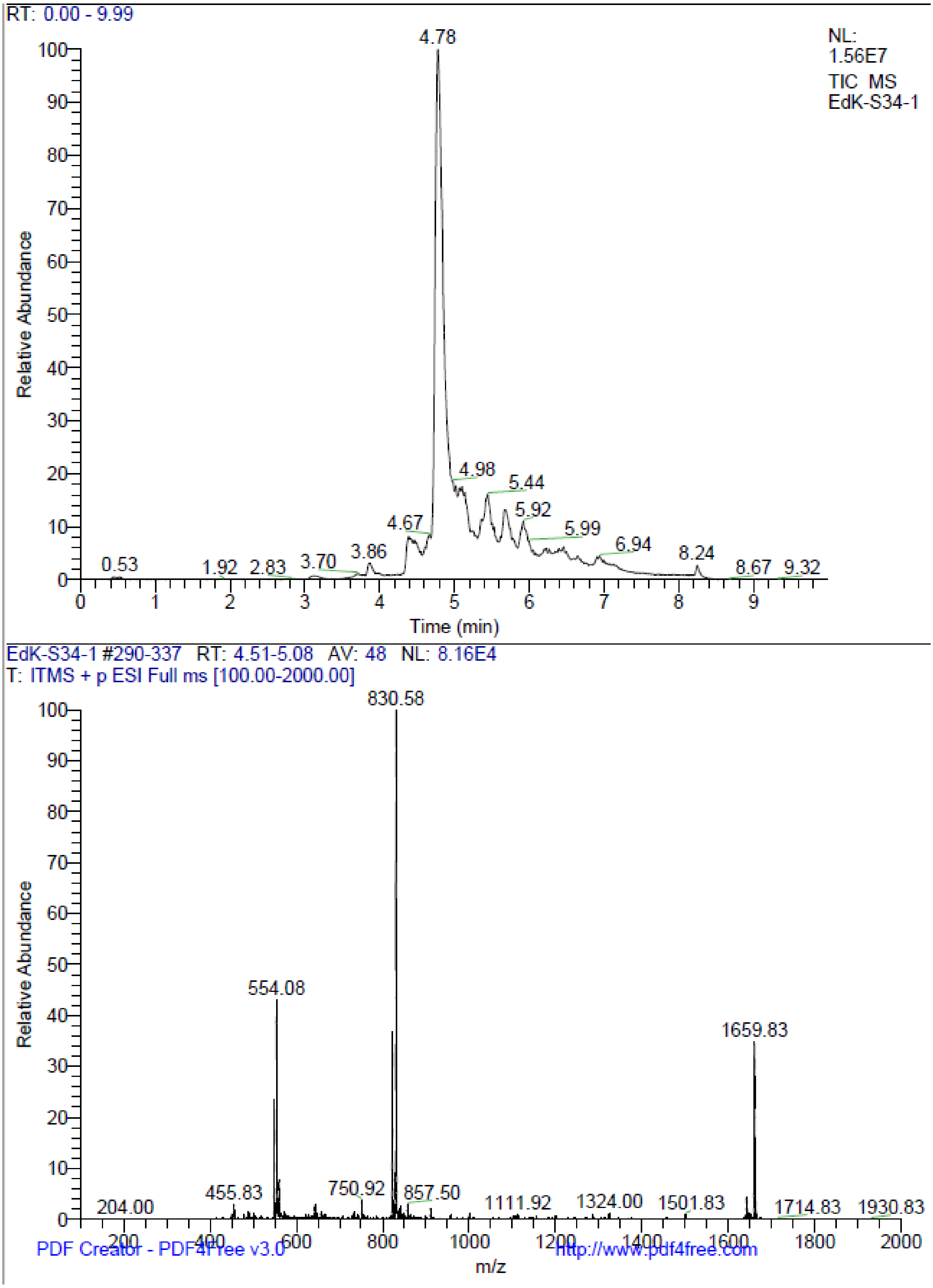

Figure S6 LC-MS of compound **1**.

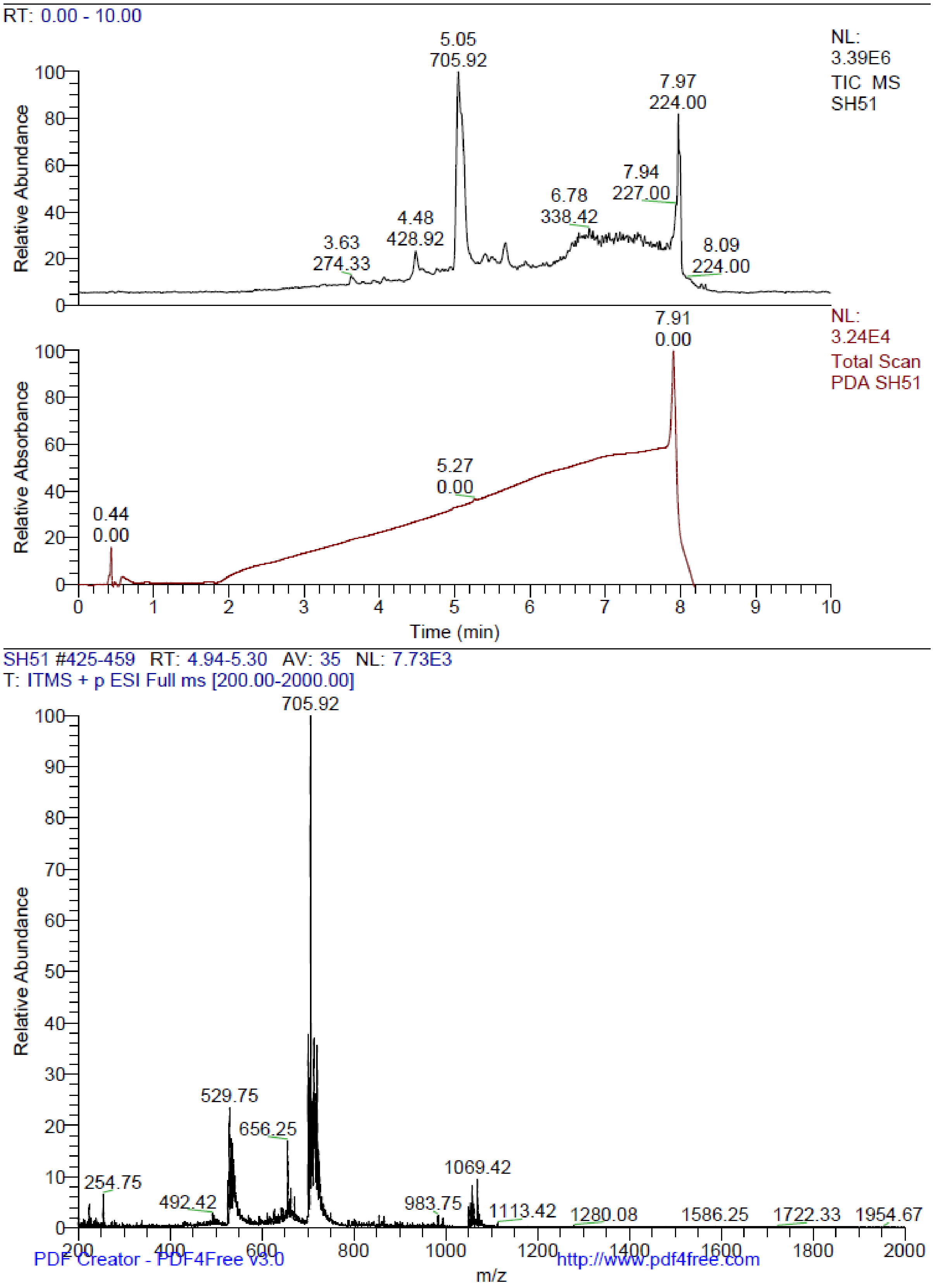

PDF Creator - PDF4Free v3.0 http://www.pdf4free.com

Figure S7 LC-MS of **BTA-Ba-Cy5**.

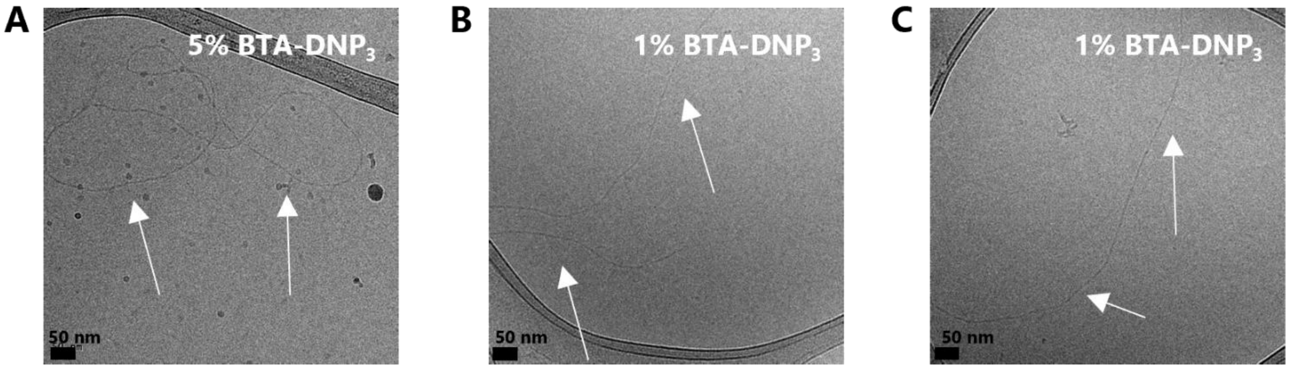

Figure S17. Cryo-TEM images upon copolymerization of **BTA-(OH)$_3$** with different mol% of DNP-functionalized monomers. ($c_{BTA,\ total}$ = 500 µM, T = 20 °C). (A) BTA-DNP$_3$ 5%, (B) BTA-DNP$_3$ 1%, (C) BTA-DNP$_3$ 1% (same sample as (B), different position on grid). Fibers are indicated by the white arrows in the cryo-TEM images.

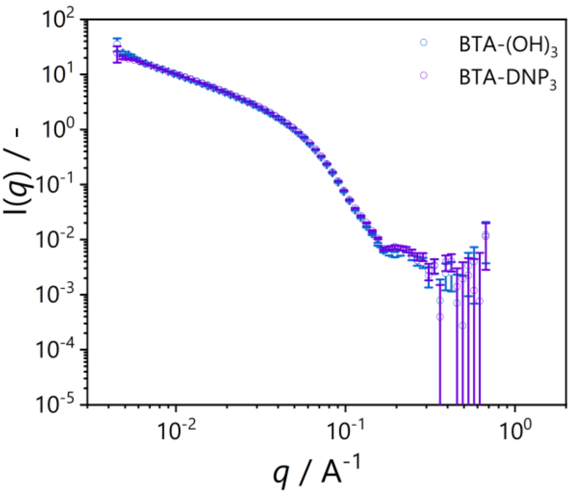

Figure S28. Overlay of SANS scattering profiles in D_2O (c = 4.5 mM).

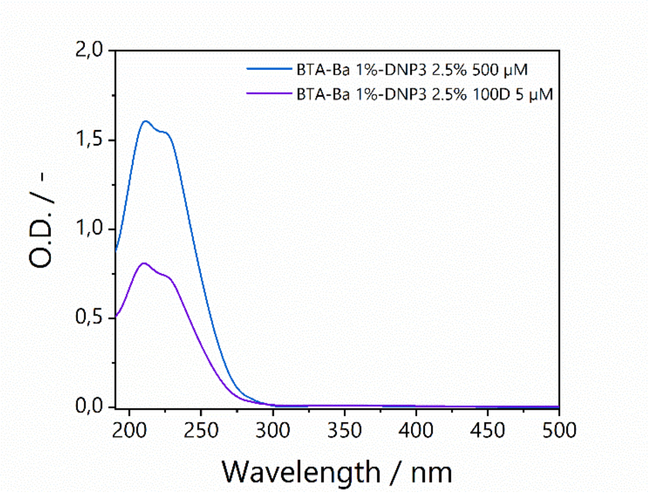

Figure S89. UV-Vis spectra of copolymers **BTA-Ba 1% BTA-DNP$_3$ 2.5%** in water before and after 100 times dilution, indicating that the nature of the supramolecular aggregates does not change. (initial c = 500 μM, after dilution 5 μM, UV-Vis spectra recorded 3 minutes after dilution, T = 20 °C. Pathlength of 1 mm (500 μM samples) or 5 cm (5 μM samples))

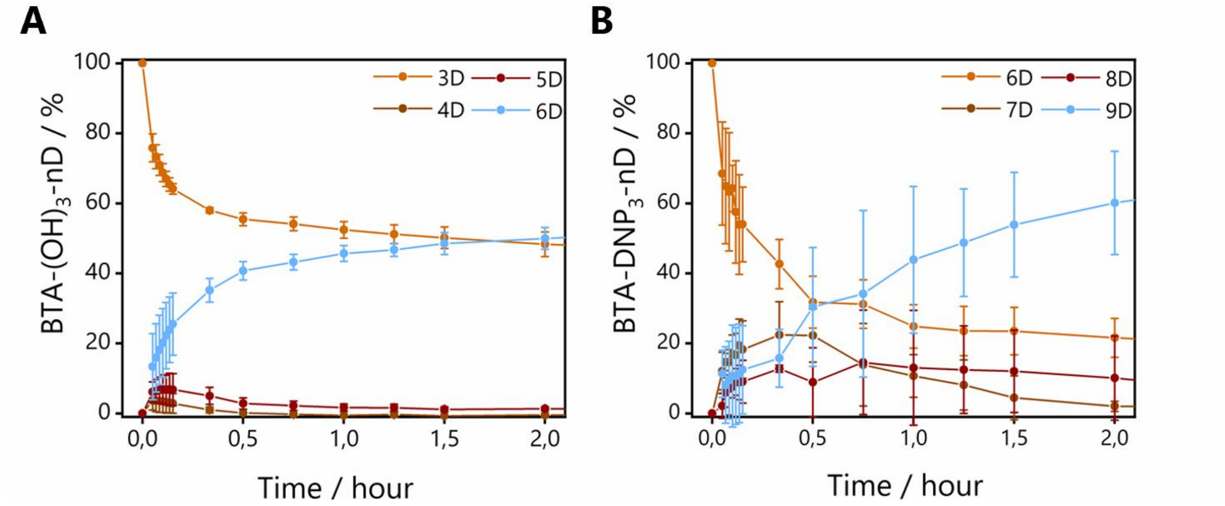

Figure S20. HDX-MS curves of after 100 times dilution of an aqueous sample into D_2O (initial *c* = 500 μM, after dilution *c* = 5 μM, T = room temperature) of **BTA-(OH)$_3$** (A) and **BTA-DNP$_3$** (B) monomers within a **BTA-DNP$_3$** : **BTA-Ba** : **BTA-(OH)$_3$** (2.5 : 1 : 96.5) copolymer The graphs show the amount of different deuterated monomers as a function of time. The error bars represent the standard deviation calculated from three separate experiments. Lines are added to guide the eye.

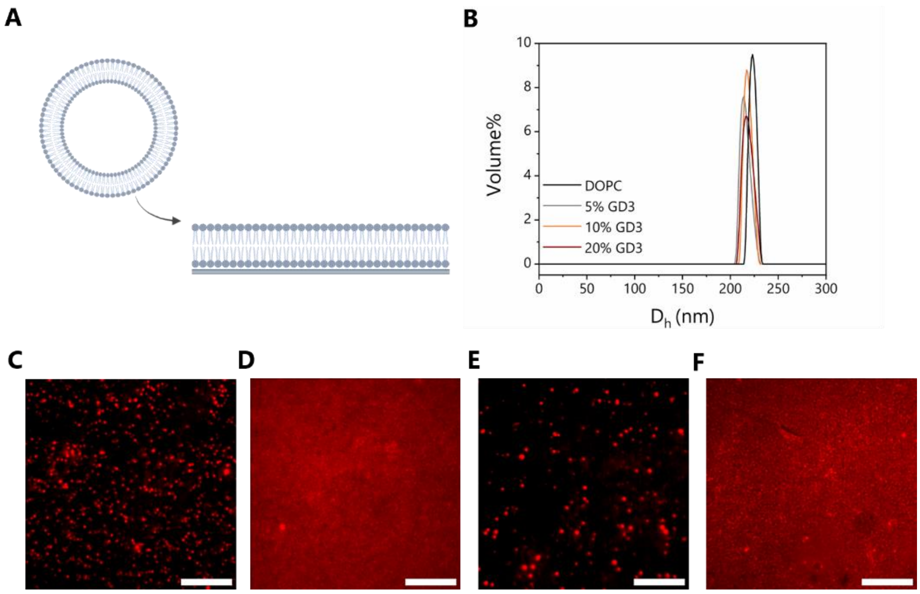

Figure S22 SLB formation. (A) Schematic representation of the vesicle fusion method. (B) DLS of vesicles composed of DOPC lipids with varying molar ratios of GD3 lipids ($c_{lipid,\ total}$ = 1 mg/mL). (C)-(F) TIRF images of SLB formation. DOPC vesicles (C) and SLB upon vesicle fusion (D), 20% GD3 vesicles (E) and 20% GD3 SLB upon vesicle fusion (F) (scalebar = 10 µm, $c_{lipid,\ total}$ = 0.1 mg/mL in phosphate buffered saline (PBS) (pH 7.4), labelled with 5 mol% NileRed)

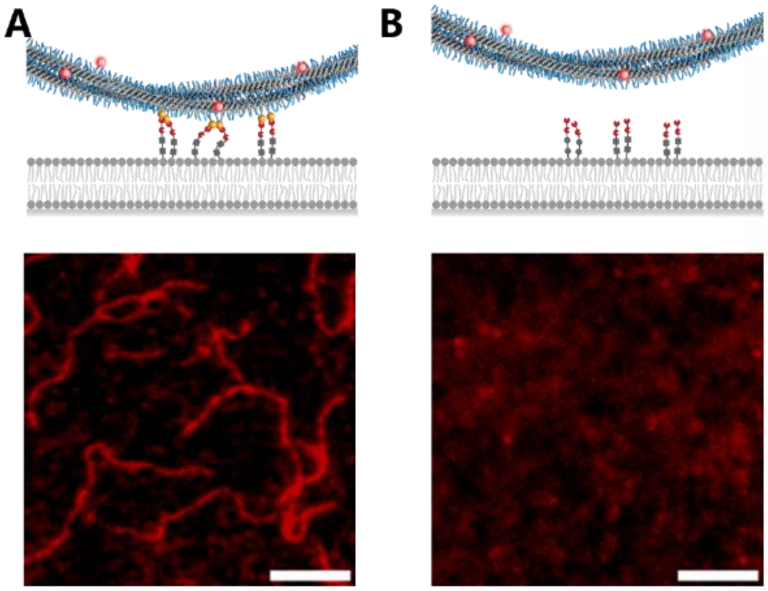

Figure S23 Interaction of BTA fibers with supported lipid bilayers. Schematic representation and TIRF image of (A) interacting fibers (**BTA-Ba** 1%) (B) non-interacting fibers (**BTA-Ba** 0%) on 20% GD3 SLB. ($c_{BTA,total}$ = 2.5 µM, **BTA-Cy5** 5%, with respect to total BTA concentration, scalebar = 5 µm).

7. References

1 L. Albertazzi, D. Van Der Zwaag, C. M. A. Leenders, R. Fitzner, R. W. Van Der Hofstad and E. W. Meijer, *Science*, 2014, **344**, 491–495.

2 G. Morgese, B. F. M. Waal, S. Varela-Aramburu, A. R. A. Palmans, L. Albertazzi and E. W. Meijer, *Angewandte Chemie*, 2020, **132**, 17382–17386.

3 M. E. J. Vleugels, S. Varela-Aramburu, B. F. M. de Waal, S. M. C. Schoenmakers, B. Maestro, A. R. A. Palmans, J. M. Sanz and E. W. Meijer, *Biomacromolecules*, 2021, **22**, 5363–5373.

4 C. M. A. Leenders, L. Albertazzi, T. Mes, M. M. E. Koenigs, A. R. A. Palmans and E. W. Meijer, *Chem. Commun.*, 2013, **49**, 1963–1965.

5 M. B. Baker, R. P. J. Gosens, L. Albertazzi, N. M. Matsumoto, A. R. A. Palmans and E. W. Meijer, *ChemBioChem*, 2016, **17**, 207–213.

6 X. Lou, S. M. C. Schoenmakers, J. L. J. van Dongen, M. Garcia-Iglesias, N. M. Casellas, M. Fernández-Castaño Romera, R. P. Sijbesma, E. W. Meijer and A. R. A. Palmans, *J. Polym. Sci.*, 2021, **59**, 1151–1161.

7 D. A. Case, R. M. Betz, D. S. Cerutti, T. E. Cheatham, T. A. Darden, R. E. Duke, T. J. Giese, H. Gohlke, A. W. Goetz, N. Homeyer, S. Izadi, P. Janowski, J. Kaus, A. Kovalenko, T. S. Lee, S. LeGrand, P. Li, C. Lin, T. Luchko, R. Luo, B. Madej, D. Mermelstein, K. M. Merz, G. Monard, H. Nguyen, H. T. Nguyen, I. Omelyan, A. Onufriev, D. R. Roe, A. Roitberg, C. Sagui, C. L. Simmerling, W. M. Botello-Smith, J. Swails, R. C. Walker, J. Wang, R. M. Wolf, X. Wu, L. Xiao and P. A. Kollman, P. A. AMBER 12; University of California: San Francisco, CA, 2012.

8 J. Wang, R. M. Wolf, J. W. Caldwell, P. A. Kollman and D. A. Case, *J Comput Chem*, 2004, **25**, 1157–1174.

9 A. Jakalian, B. L. Bush, D. B. Jack and C. I. Bayly, *J Comput Chem*, 2000, **21**, 132–146.

10 A. Jakalian, D. B. Jack and C. I. Bayly, *J Comput Chem*, 2002, **23**, 1623–1641.

11 M. D. Hanwell, D. E. Curtis, D. C. Lonie, T. Vandermeersch, E. Zurek and G. R. Hutchison, *J Cheminform*, 2012, **4**:17.

12 G. D. Hawkins, C. J. Cramer and D. G. Truhlar, *Chem Phys Lett*, 1995, **246**, 122–129.

13 D. S. Cerutti, R. Duke, P. L. Freddolino, H. Fan and T. P. Lybrand, *J Chem Theory Comput*, 2008, **4**, 1669–1680.

14 W. Humphrey, A. Dalke and K. Schulten, *J Mol Graph*, 1996, **14**, 33–38.

15 W. L. Delano, Tne PyMOL Molecular Graphics System, Version 1.8.4.0 Schrödinger, LLC.

16 D. R. Roe and T. E. Cheatham, *J Chem Theory Comput*, 2013, **9**, 3084–95.
